# Distinctive actions of connexin 46 and connexin 50 in anterior pituitary folliculostellate cells

**DOI:** 10.1371/journal.pone.0182495

**Published:** 2017-07-31

**Authors:** María Leiza Vitale, Christopher J. Garcia, Casimir D. Akpovi, R.-Marc Pelletier

**Affiliations:** Département de pathologie et biologie cellulaire, Faculté de Médecine, Université de Montréal, Montreal, Québec, Canada; Universidad de Salamanca, SPAIN

## Abstract

Folliculostellate cell gap junctions establish a network for the transmission of information within the anterior pituitary. Connexins make up gap junction channels. Changes in connexin (Cx) turnover modify gap junction-mediated intercellular communication. We have reported that cytokines and hormones influence Cx43 turnover and coupling in folliculostellate cells and in the folliculostellate cell line TtT/GF. In addition, the expression of different connexins alters intercellular communication and connexins may have functions besides cell coupling. Here we assessed the expression, turnover and subcellular localization of Cx46 and Cx50 in the anterior pituitary and TtT/GF cells. Then, we assessed the impact of various natural (lactation, annual reproductive cycle, bFGF) and pathological (autoimmune orchitis, diabetes/obesity) conditions associated with altered anterior pituitary hormone secretion on Cx46 and Cx50. Anterior pituitary Cx46 and Cx50 expression and subcellular distribution were cell-dependent. Cx46 was expressed by folliculostellate, TtT/GF and endocrine cells. In the cytoplasm, Cx46 was chiefly associated with lysosomes. Variously sized Cx46 molecules were recovered exclusively in the TtT/GF cell nuclear fraction. In the nucleus, Cx46 co-localized with Nopp-140, a nucleolar factor involved in rRNA processing. Neither cytoplasmic nor nuclear Cx46 and Cx43 co-localized. Cx50 localized to folliculostellate and TtT/GF cells, and to the walls of blood capillaries, not to endocrine cells. Cx50 was cytoplasmic and associated with the cell membrane, not nuclear. Cx50 did not co-localize with Cx46 but it co-localized in the cytoplasm and co-immunoprecipitated with Cx43. Cx46 and Cx50 responses to various physiological and pathological challenges were different, often opposite. Cx46 and Cx43 expression and phosphorylation profiles differed in the anterior pituitary, whereas Cx50 and Cx43 were similar. The data suggest that Cx46 participates to cellular growth and proliferation and that Cx50, together with Cx43, contributes to folliculostellate cell coupling.

## Introduction

The folliculostellate (FS) cells together with endocrine cells constitute the anterior pituitary gland parenchyma. The FS cells control several anterior pituitary activities [1]. Specifically, FS cells produce cytokines and growth factors that regulate anterior pituitary hormone secretion [2;3]. At variance with the anterior pituitary endocrine cells, FS cells contain no secretory granules [4] and express the protein S-100 [5]. In addition, by enclosing endocrine cells in clusters, the FS cell cytoplasmic processes organize the anterior pituitary parenchyma into follicles [6–8]. We and others have shown that the extent of FS cell cytoplasmic processes is responsive to the hormonal milieu [8;9] and to serum-borne molecules [10] such as the basic fibroblast growth factor (bFGF) [11;12].

The gap junction-mediated cell-to-cell communication allows the sharing of information and thus, the coordination and synchronization of responses in connected cells. Within the anterior pituitary, gap junctions join FS-to-FS cells [7;13–15], FS cells-to-endocrine cells [16–18] and endocrine cells-to-endocrine cells [19]. Experimental evidence shows that regulators of anterior pituitary function modulate FS cell connectivity by acting on FS cell gap junctions [12;15;20–25].

The gap junction channels are made up of proteins named connexins (Cxs). The rodents and human have been found to express approximately 20 Cx variants classified into subgroups based on sequence homology and oligomerization [26;27]. The regulation of gap junction-mediated intercellular communication is achieved by modifying Cx turnover [28] and/or the expression of individual Cx species [29]. Cx43 is expressed by the FS cells [15;17;30] and the cells of the FS cell line TtT/GF [23]. Here, we assessed the expression of Cx46 and Cx50, two α-Cxs known to interact with Cx43 [26], in anterior pituitary FS cells and TtT/FG cells. Cx46 and Cx50 have been extensively studied in the ocular lens [31]. In addition, bone [32–34], lung [35], retinal pigmented epithelial cells [36], heart [37], astrocytes [38] and human breast tumour [39] all express Cx46. Cx50 expression has been described in the retina [40] and corneal endothelial cells [41]. Recently, we reported the expression of Cx46 and Cx50 in cells of the developing and adult testes [42].

Cxs are critically involved in strategic steps of tissue and cell actions. Mutated *Cx* genes, deregulation of Cx turnover, and/or aberrant localization of Cxs have been documented in pathological disorders [43–46]. Cxs also contribute to other cellular functions besides cell coupling [43;47;48]. To evaluate Cx46 and Cx50 involvement in the anterior pituitary function, the behavior of Cx46 and Cx50 was assessed in physiological and pathological conditions that display changes in anterior pituitary hormone secretion. Specifically, the annual seasonal reproductive cycle offers a unique opportunity to evaluate the influence of *natural* and reversible hormonal changes on the expression, phosphorylation status and localization of Cx46 and Cx50 in the anterior pituitary. We have established the mink (*Mustela vision*) as a valuable model for the study of FS cell physiology and Cx43 turnover and distribution during periods of increased prolactin (Prl) secretion specifically, lactation [8;15]. Moreover, we have shown that in the male mink, the natural seasonal modulation of anterior hormone secretion is during spontaneous autoimmune orchitis (AIO) [49;50]. Next, we investigated the behavior of Cx46 and Cx50 in a third model, the *ob/ob* leptin-deficient and the *db/db* leptin receptor-deficient male mice which exhibit abnormal anterior pituitary hormone levels [51–53]. The *db/db* and the *ob/ob* mice are diabetic, obese and infertile. Diabetes and obesity have been shown to modify Cx levels in blood vessels and other tissues in the body [46;54]. Moreover, leptin, known chiefly for its food intake inhibition virtues also impacts anterior pituitary hormone secretion [51;55] and FS cell gap junctions [22]. The fourth model used in the present investigation is the FS cell line TtT/GF [11]. We have established the TtT/GF cell line as model to evaluate the action of cytokines and growth factors on FS cell coupling and Cx43 turnover [12;23;24]. We took advantage of the FS cell line TtT/GF model to determine the influence of bFGF on the Cx46 and Cx50 behavior.

The results show significant differences in the distribution of Cx46 and Cx50 within the anterior pituitary, the FS and TtT/GF cells. Moreover, Cx46 and Cx50 exhibited distinct and often opposite responses to the various physiological and pathological challenges they were exposed. The results suggest that, together with Cx43, Cx50 participates in cell-to-cell communication and that Cx46 may contribute to the proliferation and growth of FS cells.

## Materials and methods

### Ethics statement

Mice were first anaesthetized with urethane before being decapitated. The protocol was approved by the « Comité de déontologie de l’expérimentation sur les animaux » of the Université de Montréal (Permit number 12–126). Mink were anaesthetized using sodium pentobarbital (0.2 ml/kg), then decapitated. The protocol was approved by the « Comité de déontologie de l’expérimentation sur les animaux » of the Université de Montréal (Permit number 15–108).

### Source of antibodies, cytokines and other compounds

The antibodies used in the present study are listed in Table 1. Three different Cx46 antibodies all raised against the C-terminal region of Cx46 were used. A rabbit polyclonal IgG from Alpha Diagnostic Intl. Inc. (San Antonio, TX, USA), a rabbit polyclonal IgG from Invitrogen Canada Inc. (Burlington, ON, Canada) and a rabbit polyclonal IgG from U.S. Biological (Salem, MA, USA). Two different Cx50 antibodies were used, a mouse monoclonal IgM (Invitrogen Canada Inc.) and a rabbit polyclonal IgG (Alpha Diagnostic Intl. Inc.). Earlier, we have established the specificity of the antibodies in tissues from Cx46- and Cx50-knock out mice and mink lens [42]. Rabbit polyclonal anti-Cx43, rabbit polyclonal anti-actin, mouse monoclonal anti-actin, protein A Sepharose-4B, phosphatase and protease inhibitors and general chemicals were purchased from Sigma Chemical Co (Windsor, ON, Canada). Mouse monoclonal antibodies against Cx43 and Promyelocytic leukemia (PML) protein were from Millipore (Billerica, MA, USA). Rabbit polyclonal IgG against calnexin was from Calbiochem (San Diego, CA, USA). Rabbit polyclonal antibodies against caveolin-1 and early endosome antigen-1 (EEA-1), and mouse monoclonal antibodies against Nopp-140 and p80/coilin were from Santa Cruz Biotech. (Dallas, TX, USA). Mouse monoclonal anti-GM130 and mouse monoclonal anti flotillin-1 were from BD Transduction Labs. (Missisauga, ON, Canada). Mouse monoclonal anti trans-Golgi TGN-38 was purchased from Affinity Bioreagents (Golden, CO, USA). The antibody against LAMP1 (1D4B), developed by Dr. Thomas August, was obtained from the Developmental Studies Hybridoma Bank University of Iowa, Dept Biological Sciences developed under the auspices of the NICHD. Rabbit anti-rat prolactin (NIDDK-anti-rPRL-IC-5) was from National Institutes of Health (Bethesda, MD, USA). Rabbit polyclonal IgG against glyceraldehyde-3-phosphate dehydrogenase (GAPDH) was from Abcam Inc. (Toronto, ON, Canada). Horseradish-peroxidase (HRP)-, biotinylated-, fluorescein isothiocyanate (FITC)-, and tetramethylrhodamine isothiocyanate (TRITC)-conjugated secondary antibodies were from Jackson Immmunochemicals (West Grove, PA, USA) or Sigma Chemical Co. bFGF was obtained from Biosource (Camarillo, CA, USA). TRITC-conjugated Concanavalin-A, TRITC-conjugated Wheat Germ Aglutinin (WGA), and HRP-conjugated streptavidin were from Molecular Probes (Eugene, OR, USA). Alkaline phosphatase, aprotinin, phenylmethylsulfonyl fluoride (PMSF) and chemiluminescence detection kit Lumilight^™^ were from Roche (Laval, QC, Canada). Proteins were measured by the Bradford dye binding assay (BioRad, Mississauga, ON, Canada). Materials for cell culture were purchased from GIBCO (Burlington, ON, Canada).

**Table 1:** List of primary antibodies and probes.

Antibody or probe	Isotype	Associated sub-cellular structure	Dilutions		
			WB	IF	IHC
Calnexin	Rabbit polyclonal IgG	Endoplasmic reticulum	1/3000	1/250	N/A
Caveolin-1	Rabbit polyclonal IgG	Caveolae	1/5000	1/50	N/A
Coilin/p80	Mouse monoclonal IgG	Cajal bodies	1/250	1/2	N/A
Concanavalin A	Probe	Endoplasmic reticulum	N/A	1/150	N/A
Connexin 43 (GJA1)	Mouse monoclonal IgG	Gap junction	1/200	1/7	N/A
	Rabbit polyclonal IgG		1/18000	1/200	N/A
Connexin 46 (GJA3)	Rabbit polyclonal IgG (Alpha Diag.)	Gap junction	1/250	1/5	1/200
	Rabbit polyclonal IgG (Invitrogen)		1/250	1/10	N/A
	Rabbit polyclonal IgG (US Biologicals)		1/50	N/A	N/A
Connexin 50 (GJA8)	Mouse monoclonal IgM	Gap junction	1/1000	1/10	1/500
	Rabbit polyclonal IgG		1/500	N/A	N/A
EEA-1	Goat polyclonal IgG	Early endosomes	N/A	1/5	N/A
Flotillin-1	Mouse monoclonal IgG	non caveolar-lipid rafts	1/500	1/5	N/A
GAPDH	Rabbit polyclonal IgG	Glycolytic pathway	1/1000	N/A	N/A
GM-130	Mouse monoclonal IgG	*cis*-Golgi	1/500	1/25	N/A
LAMP-1	Rat monoclonal IgG2a	Lysosomes	N/A	1/20	N/A
NOPP-140	Rabbit polyclonal IgG	Nucleolus and Cajal bodies	1/1000	1/100	N/A
	Mouse polyclonal IgG		1/250	1/2	N/A
PML	Mouse monoclonal IgG	PML bodies	1/2000	1/150	N/A
Prl	Rabbit polyclonal IgG	Prolactin granules	1/6000	N/A	N/A
TGN-38	Mouse monoclonal IgG	*trans*-Golgi	N/A	1/10	N/A
WGA	probe	Golgi apparatus	N/A	1/60	N/A

### Cell culture

Cells from the anterior pituitary FS cell line TtT/GF were initially provided by Dr. U. Renner (Max-Planck-Institute of Psychiatry, Dept. of Endocrinology, Munich, Germany). TtT/GF cells exhibit the morphological, biochemical and physiological features of typical FS cells [7;11]. The cells were grown in DMEM supplemented with 5% fetal calf serum, 3.7 g/ml NaHCO_3_, 10 mM HEPES, pH 7.2, and antibiotics at 37°C under a 95%-5% air-CO_2_ atmosphere. For immunofluorescence studies, the cells were seeded on glass coverslips (No. 0 thickness). Cells were serum-starved for 24 h prior treatment with bFGF (15 ng/ml).

Cells were harvested once they had reached 60–70% confluence. After a wash with cold phosphate buffered saline (PBS: 137 mM NaCl, 3 mM KCl, 8 mM Na_2_HPO_4_ and 1.5 mM KH_2_PO_4_, pH 7.4), the cells were detached and recovered by centrifuged at 2,000 RPM for 5 min in a Beckman, GS-6R centrifuge with a GH 3.8 rotor (Beckman Coulter Canada Inc., Mississauga, ON, Canada). The cell pellet was resuspended in a protease phosphatase inhibitor cocktail made in PBS (4mM Na_3_VO_4_, 80 mM NaF, 20 mM Na_4_P_2_O_7_, 10 μM bpV-phen, 5 μg/ml leupeptin, 5 μg/ml aprotinin and 2mM EGTA, pH 8.5), except for the alkaline phosphatase studies, and sonicated at moderate intensity when needed (Fisher Sonic Dismembranator Model 300) for 30 seconds.

### Animals and tissues

#### Mink

**Mink** (*Mustela vison*) were purchased from Visonnière St. Damase mink farm (St Damase, QC, Canada). Animals were kept under natural lighting conditions and were allowed food and water *ad libitum*. Mink were anaesthetized using sodium pentobarbital (0.2 ml/kg), then decapitated. Female mink: Lactating and non-lactating female mink were sacrificed during the lactation period (May), when prolactin (Prl) is high [15]. Normal adult mink: Tissues were collected from 2-3-year-old fertile adults in the last week of February (active phase of the annual reproductive cycle) and July (inactive phase of the annual reproductive cycle [56]). Infertile mink with autoimmune orchitis (AIO): Two to 3-years old Black and Sapphire mink that mated and sired 5 or more litters the previous year but were sterile during the current year and diagnosed with secondary infertility related to spontaneous AIO were utilized [50]. Animal protocols were conducted in conformity with the “Comité de déontologie de l’expérimentation sur les animaux” of the Université de Montréal (Permit number 15–108).

#### Mice

Male mice aged of 10 weeks with the leptin receptor (B6.BKS(D)-*Leprdb*/J homozygotes (*db*/*db*) Stock Number 00697) mutation, male mice aged of 10 weeks with the leptin (B6.Cg-*Lepob*/J homozygotes (*ob/ob*) Stock Number 00632) mutation and 10 week-old wild type (WT) mice were purchased from Jackson Lab (Bar Harbor, ME, USA) and housed at room temperature (RT) with food and water *ad libitum* and exposed to a 12 h: 12 h light-dark cycle. Mice were first anaesthetized with urethane (1 g/kg IP, Sigma, St-Louis, MO, USA) before being decapitated. Animal protocols were conducted in conformity with the “Comité de déontologie de l’expérimentation sur les animaux” of the Université de Montréal (Permit number 12–126)

#### Anterior pituitary

After decapitation, the anterior lobe of mink and mouse pituitary glands were dissected free from the intermediate and posterior lobes. For biochemical studies, anterior pituitaries were placed in PBS-protease and phosphatase inhibitors, sonicated and stored at -80°C until use [15]. For immunohistochemical studies the tissues were immersed in fixative as detailed below.

#### Mouse ocular lens

Lenses excised from normal mouse eyes were briefly immersed into dry ice then fragmented. The fragments were allowed to thaw, then homogenized in PBS-protease and phosphatase inhibitors using a Polytron PT 3100 homogenizer (Kinematica, Lucerne, Switzerland) [42]. Aliquots of the total homogenate were stored at -80°C until use.

### Preparation of crude cytosolic and membrane fractions

Isolation of the membrane- and cytosol-enriched subcellular fractions was carried out as previously described [10;57]. Briefly, cells were washed with PBS containing protein phosphatase and protease inhibitors, collected by scraping, and homogenized to obtain a total cell lysate. Twenty μl of total cell lysate were put aside and the remaining lysate was centrifuged at 1,500*g* (Beckman GS-6R; Beckman, Canada) and the pellet discarded. The supernatant (S1) was again centrifuged at 15,000*g* (Beckman TLA55; Beckman, Canada) at 4°C for 20 min. This supernatant (S2) was considered as the crude cytosol-enriched fraction. The pellet, considered as the crude membrane fraction, was rinsed in PBS buffer, resuspended in RIPA buffer (150 mM NaCl, 1% NP-40, 0.5% deoxycholic acid (DOC), 0.1% SDS, 50 mM Tris-HCl, pH 8.0) and briefly sonicated. The characterization of the subcellular fractions was carried out as detailed elsewhere [10;23].

### Preparation of the nuclear and post-nuclear fractions

We applied the protocol of Culjkovic et al. [58] with some modifications. Briefly, cells were first washed twice in ice-cold PBS then, collected by centrifugation. The cell pellet was resuspended in 1 ml of lysis buffer-B (140mM NaCl, 1.5mM MgCl_2_, 0.5% NP 40, 1 mM DTT, 10 mM Tris-HCl, pH 8.4) and cells were lysed by pipetting up and down 50x. The cell lysate was spun at 1,000*g* (Beckman, GS-6R centrifuge), 4°C for 3 min. The “cytoplasmic/plasma membrane” fraction, contained in the supernatant was transferred to a separate tube and resuspended in 1 ml of PBS-protease phosphatase inhibitor solution. Intact nuclei forming the pellet were resuspended in 1 ml lysis buffer-B and transferred to a round bottom tube. Under slow vortexing, 100 μl of detergent stock (3.3% (w/v) DOC and 6.6% (v/v) Tween^®^ 40) was added to the resuspended pellet which was then incubated on ice for 5 min. Following incubation, the nuclear fraction was spun at 1,000*g*, 4°C for 3 min (Beckman, GS-6R). The supernatant was added to the previously-isolated “cytoplasmic/plasma membrane” fraction. The pellet, containing a purified nuclear fraction, was rinsed in 1 ml of lysis buffer-B then spun at 1,000*g*, 4°C for 3 min (Beckman, GS-6R). The supernatant was discarded and the pellet, consisting of intact purified nuclei, was collected. Throughout the purification steps, preparations were visually assessed under light microscopy. The intact nuclei were resuspended in a PBS-protease phosphatase inhibitor solution then, lysed by sonication (30 seconds at moderate intensity). The protein content of the whole cell lysate, the cytoplasmic/plasma membrane (post-nuclear) fraction and the nuclear lysate was determined and samples were prepared for electrophoresis.

### Electrophoresis and Western blot

Ten-thirty μg proteins were loaded onto 10–12% SDS-polyacrylamide gels. After electrophoresis, the proteins were transferred onto nitrocellulose membranes (Bio-Rad, Mississauga, ON, Canada). The membranes were quickly stained with Ponceau red to ascertain equal loading, rinsed with PBS, blocked with 5% skimmed milk in PBS and incubated with the first antibody (Table 1). Following extensive rinsing, the membranes were incubated with the corresponding second antibody coupled to HRP (1/2,000); next, they were stripped and reprobed with either monoclonal anti-actin (1/2,000) or polyclonal anti-actin (1/3,000). Bands on the films were scanned and their intensities were quantified by using the Scion Image Program (Scion Corporation, MD, USA). The immunoreactive band intensity values were normalized to the corresponding actin band intensity value as previously described [12;42].

### Alkaline phosphatase treatment

Studies on the phosphorylation of Cx46 were carried out as described elsewhere [42] with some modifications. Briefly, TtT/GF cells were collected by scraping and rinsed in PBS without phosphatase inhibitors. Excess PBS was removed and cold digestion buffer (10 mM MgCl_2_, 1 mM ZnCl_2_, 50 mM Tris-HCl, pH 8.0) was added to the pellet, which was resuspended, then sonicated. One hundred μg of TtT/GF lysate protein samples were prepared. Thirty units of alkaline phosphatase were added to each sample (+APh), while equal volumes of digestion buffer were added to control samples (-APh). Then, samples were incubated 30 min in a 37°C water bath under gentle agitation. Alkaline phosphatase activity was halted with PBS-protease phosphatase inhibitor and the samples were placed on ice for 15 min. Thirty μg proteins per sample were subjected to SDS-PAGE followed by Western blotting Cx46 antibodies.

### Co-immunoprecipitation

Co-immmunoprecipitation studies were carried out as described earlier [59]. Briefly, whole TtT/GF cell lysates were centrifuged for 20 min at 15,000*g* (Beckman microfuge E), and 1 ml of supernatant was incubated with 100 μl of protein A Sepharose bead slurry (50%) at 4°C for 1 hour on a rocker to pre-clear the cell lysates. Protein A Sepharose beads were removed by centrifugation at 15,000*g* at 4°C for 10 min. Pre-cleared supernatants (2 mg/ml) were incubated overnight at 4°C with either buffer (control) or rabbit poly-clonal anti-Cx43. One hundred μl of protein A Sepharose 4B beads, pre-washed with PBS, were added to each sample and the mixtures were further incubated for 4 hours at 4°C. The beads were recovered by centrifugation at 15,000*g*, rinsed several times in lysis buffer, and subjected to SDS-PAGE and Western blotting with either mouse anti-Cx50 or mouse anti-Cx43.

### Immunohistochemistry

Mink anterior pituitaries were immersion-fixed in Bouin’s solution for 1h at RT with continuous agitation immediately after dissection [8]. Potential endogenous peroxidase activity was inhibited with 0.6% H_2_O_2_ in Tris-buffered saline (TBS: 140mM NaCl, 50mM Tris-HCl, pH 7.4) for 10 min [8]. Anterior pituitary sections obtained under different experimental conditions were mounted sequentially on the same glass slide and simultaneously subjected to the same methodological and technical conditions. Sections were washed in TBS containing 0.1% Tween^®^ 20 (TBST) [60] and incubated for 60 min at 37°C with 1% skimmed milk in TBST to block unspecific labelling and incubated overnight at RT with anti-Cx46 or anti-Cx50 (Table 1) and next with biotinylated anti-rabbit IgG or biotinylated anti-mouse IgM for 60 min followed by HRP-conjugated streptavidin. Sections were washed in TBST and incubated for 10 min at RT in 0.01% H_2_O_2_, 0.05% diaminobenzidine tetrachloride (DAB) and 10mM imidazole in TBS (pH 7.7) [61] and mounted with Permount. Negative controls included the omission of the first or second antibodies. Pictures were taken with Kodak Technical Pan films.

### Fluorescence microscopy

Cells were fixed and permeabilized using one of two methods. Method 1: Formaldehyde fixation and acetone permeabilization. Cells were fixed with 3.7% formaldehyde and permeabilized in acetone as described previously [15]. Method 2: Formaldehyde fixation and methanol permeabilization was used to better visualize nuclear proteins. Cells were fixed in 3.7% formaldehyde then permeabilized in a -20°C methanol bath for 10 min. Both methods: Following fixation and permeabilization cells were washed several times with PBS to remove any residual acetone or methanol. Non-specific binding sites were blocked using 3% skimmed milk (made in PBS Tween^®^ 20 (0.05%)) for 1 hour at RT. Cells were then incubated at 37°C for 1 hour with a probe for a cellular region or organelle and/or with primary antibodies diluted in 1% skimmed milk (made in PBS Tween^®^ 20 (0.05%)) (Table 1). Next, cells were washed with PBS and probed with FITC- or TRITC-conjugated secondary antibodies in 1% skimmed milk (1/200) for 1 hour at 37°C. The coverslips were mounted onto glass slides using Mowiol^®^ (EMD Biosciences, San Diego, CA, USA). Labelled cells were viewed with a Zeiss Axioskop 2 fluorescence microscope (Carl Zeizz Canada Ltd, Toronto, ON, Canada) and image capture was carried out with Northern Eclipse software (Empix Imaging Inc., Mississauga, ON, Canada). Double-labelled cells were viewed with a Leica DM IRB confocal microscope (Leica Microsystems Inc., Richmond Hill, ON, Canada). Preparations were visualized in successive sections throughout the Z-plane of the cells and images were captured using a Leica Confocal software. Confocal microscopy images shown correspond to a focal plane of 0.7 μm thickness.

### Statistical analyses

Analyses were done with Stata software (Stata Corporation, College Station, TX, USA). The data were evaluated with the Student’s *t* test or the analysis of variance (ANOVA) followed by the Tukey honestly significant difference test (THSDT).

## Results

### Expression and distribution of Cx46 and Cx50 in the anterior pituitary and TtT/GF FS cell line

#### Cx46 and Cx50 expression and phosphorylation status

**Fig 1:**
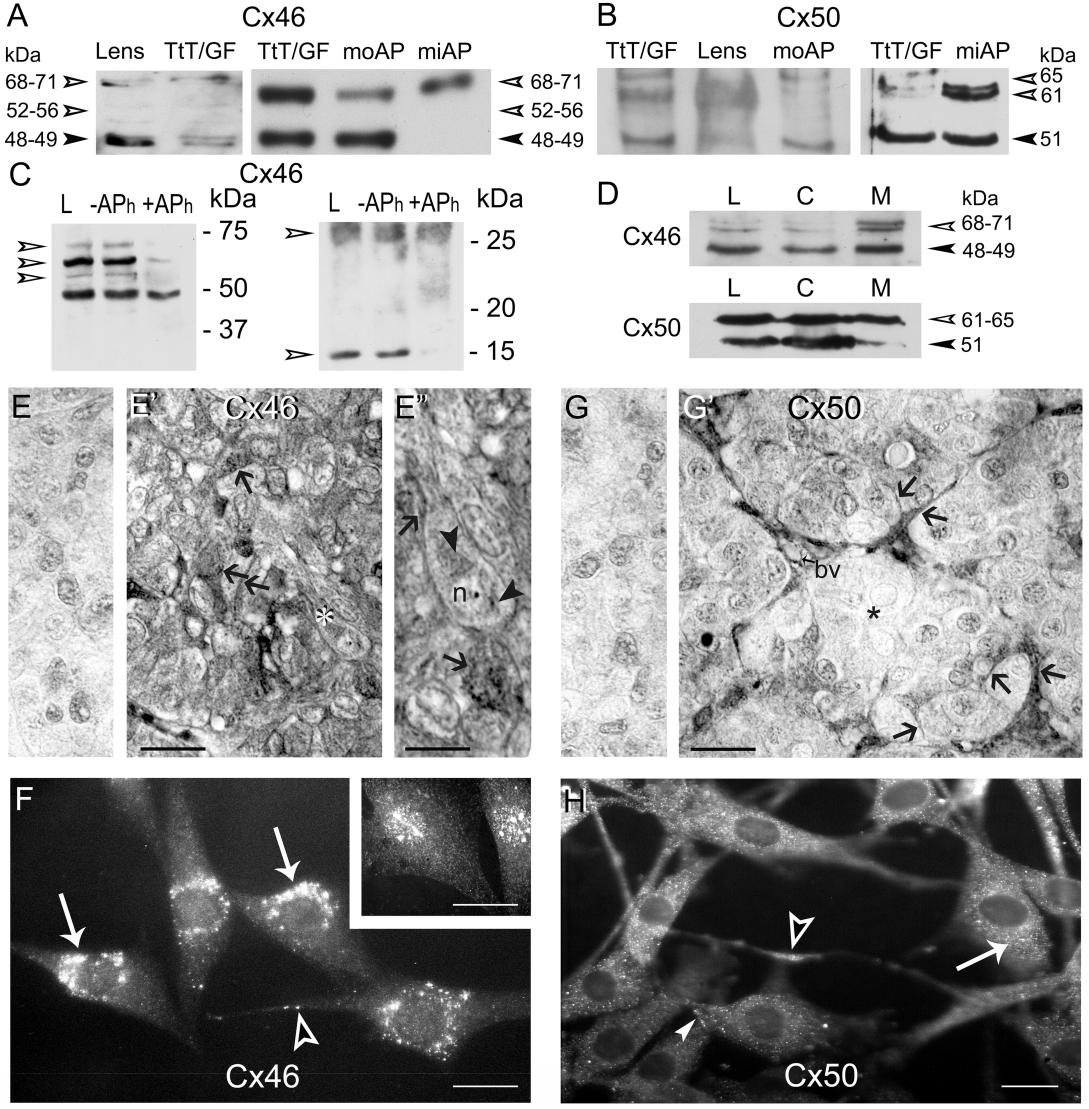
Expression, phosphorylation status and localization of Cx46 and Cx50 in TtT/GF cells and mouse and mink anterior pituitaries. (A) Left panel: lysates of mouse lens (30 μg) and TtT/GF cells (10 μg), and right panel: lysates of TtT/GF cells (5 μg) and mouse (moAP, 30 μg) and mink (miAP normal male, 20 μg) anterior pituitaries were subjected to SDS-PAGE followed by Western blotting with Cx46 and Cx50 antibodies. Cx46 antibodies detected a 48–49 kDa (arrowhead) band and a 68–71 kDa band (open arrowheads, 71 kDa) in lens. TtT/GF cells, moAP and miAP lysates exhibit the same bands with different intensities. In addition, two faint bands of molecular masses of 52, and 56 kDa were sometimes observed in lens and TtT/GF cells (open arrowheads). (B) Cx50 antibodies revealed a 51 kDa band (arrowhead) and 61 kDa band (open arrowhead) in the lens, TtT/GF cells, moAP and miAP. A third 65 kDa band was detected in TtT/GF cells and in AP lysates (open arrowhead). (C) Cx46 phosphorylation status: TtT/GF cell lysates (control: L) were incubated either in the absence (-APh) or presence (+APh) of alkaline phosphatase. Following incubation, 30 μg protein aliquots from each sample were subjected to SDS-PAGE followed by Western blotting with anti-Cx46. The left panel shows a strong ~68 kDa band flanked by ~71, 56 and 52 kDa bands whose intensities were diminished by the APh treatment (open arrowheads). The right panel shows two Cx46 immunoreactive bands at 25 and 14 kDa in whole cell lysates (L) and in lysates incubated with buffer alone (-APh). The 14 kDa band intensity was reduced by phosphatase treatment (+APh, open arrowhead). (D) TtT/GF cell lysate (L, 30 μg), cytosolic (C, 30 μg) and crude membrane (M, 30 μg) fractions were subjected to SDS-PAGE and immunoblotting with Cx46 and Cx50 antibodies. Representative Western blots show enrichment in the high molecular weight Cx46 and Cx50 immunoreactive bands in the crude membrane fraction (open arrowheads). (E-E”) Cx46 immunohistochemistry in mink AP. (E) No reaction was detected in Cx46 controls done on normal adult male mink APs incubated with either the primary or secondary antibody. (E’) The FS cell delicate cytoplasmic processes were Cx46-positive (arrows). (E”) A higher magnification of a hormone secreting cell labelled with a white asterisk in E’ displays Cx46 labelling (arrowheads) in the perinuclear region and the nucleolus (n). In (E”), the lower arrow points to heavy Cx46 labeling within the cytoplasm of an FS cell; the upper arrows points to Cx46 labelled FS cell thin cytoplasmic process surrounding an adjacent endocrine cell. E and E’, bar: 40 μm. E”, bar: 15 μm. (F) Cx46 immunofluorescence in TtT/GF cells. Cx46 staining showed a punctate, cytoplasmic distribution concentrated in the perinuclear region (arrows). Cx46 labelled cytoplasmic processes (open arrowhead). When changing the focal planes a nuclear distribution became visible (insert). F and insert, bar: 20 μm. (G-G’) Cx50 immunohistochemistry in adult male mink anterior pituitaries. (G) Cx50 controls with either the primary or secondary antibody showed no immunolabeling in adult male mink. (G’) At the periphery of the follicle, the FS cells and their delicate cytoplasmic processes adjoining endocrine cells were Cx50-positive (arrows) in contrast to the hormone secreting cells that were not (asterisk). In addition, Cx50 labeled the wall of blood vessels (bv). G and G’, bar: 40 μm. (H) Cx50 fluorescence microscopy studies in TtT/GF cells. A dust-like Cx50 labelling was evenly distributed in the cytoplasm (arrow). In addition, cytoplasmic processes (open arrowhead) and the cell membrane (arrowhead) were Cx50-positive. No immunoreactivity was apparent in the nucleus. Bar: 20 μm.

Cx46 and Cx50 protein expression was assessed in TtT/GF cells and in mouse and mink anterior pituitaries. We have established earlier the specificity of the Cx46 and Cx50 antibodies in mouse and mink tissues [42]. All the antibodies used gave the same results. Immunoblots shown were performed with antibodies from Alpha-Diagnostic or Invitrogen. Proteins from mouse lens probed with Cx46 antibodies generated an immunoreactive band around 48 kDa, that can be resolved into a 48 kDa band and a 49 kDa band when the proteins were allowed to migrate longer, and a 68 kDa band also at times viewed as a doublet (68–71 kDa) (Fig 1A). In addition, two faint 52 and 56 kDa bands were detected (Fig 1A). Similar bands were observed in TtT/GF cells. Immunoblots of mouse anterior pituitary lysates showed two strong bands at 48 kDa and a 68–71 kDa (Fig 1A). The mink anterior pituitary exhibited an intense 68–71 kDa band; when membranes were exposed longer a faint 48–49 kDa Cx46 immunoreactive band could be observed (Fig 1A), confirming our earlier report in mink testis [42]. Western blots performed with antibodies against Cx50 detected a 51 kDa band and a 61–65 kDa band in the mouse lens, TtT/GF cells and mouse and mink anterior pituitaries (Fig 1B). Earlier we showed that the 68 kDa Cx46 and 61–65 kDa Cx50 immunoreactive bands are phosphorylated forms of the proteins [42]. Here, we characterized the 52, 56 and 71 kDa Cx46 immunoreactive bands. The incubation of TtT/GF cell lysates with alkaline phosphatase (APh) revealed that the 52, 56 and 71 kDa bands were phosphorylated forms of Cx46 (Fig 1C, left panel). The dephosphorylation of Cx46 was accompanied by its rapid degradation (Fig 1C) as we described earlier in the testis [42]. More importantly, exposure of the lower portion of the membranes to Cx46 antibodies revealed a broad ~25 kDa band and an intense 14 kDa band in the control lane (Fig 1C, right panel). APh treatment decreased the intensity of the 14 kDa without affecting that of the 25 kDa band indicating that the 14 kDa band contains Cx46 phosphorylated fragments (Fig 1C). Whether the broad 25 kDa band contains phosphorylated Cx46 fragments unaffected by the APh treatment cannot be ruled out. Our subcellular fractionation studies demonstrated that the TtT/GF cell crude membrane fraction was enriched in Cx46 and Cx50 phosphorylated forms of higher molecular mass (Fig 1D).

#### Distribution of Cx46 and Cx50 in the anterior pituitary and TtT/GF cells

**Fig 2:**
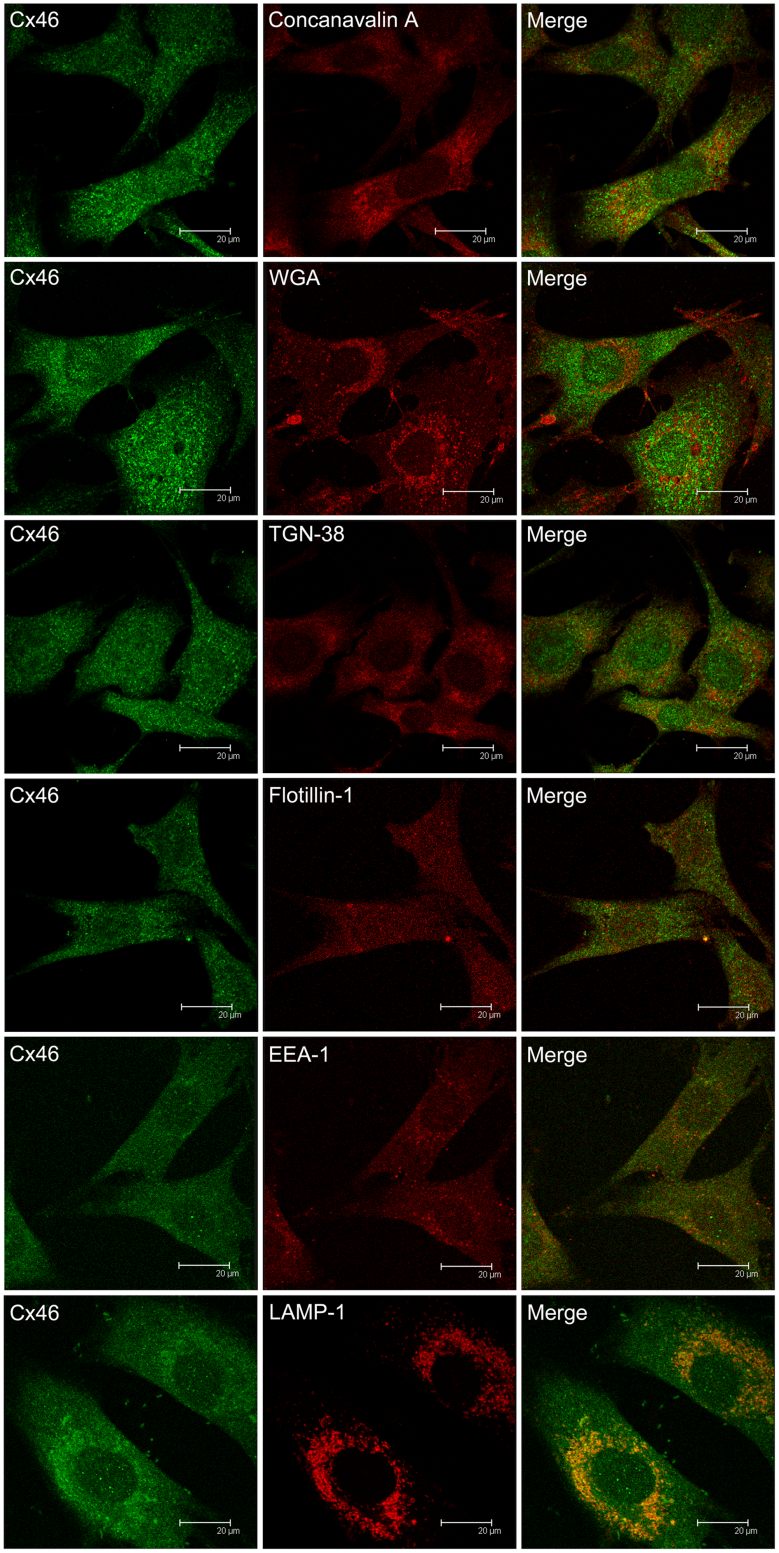
Confocal microscopy on the co-localization of Cx46 and cellular organelles in TtT/GF FS cells. TtT/GF cells were double-stained with antibodies against Cx46 and an organelle marker (antibody or probe, Table 1). Confocal microscopy images shown correspond to a focal plane of 0.7 μm thickness. Double labelling for Cx46 and either Concanavalin A (RER), TGN-38 (*trans*-Golgi network) or flotillin-1 (lipid rafts) revealed no co-localization. Cx46 co-localization with WGA (Golgi apparatus) or with EEA-1 (early endosomes) was observed in some perinuclear vesicles. A very strong Cx46 and LAMP-1 (lysosomes) co-localization was apparent.

**Fig 3:**
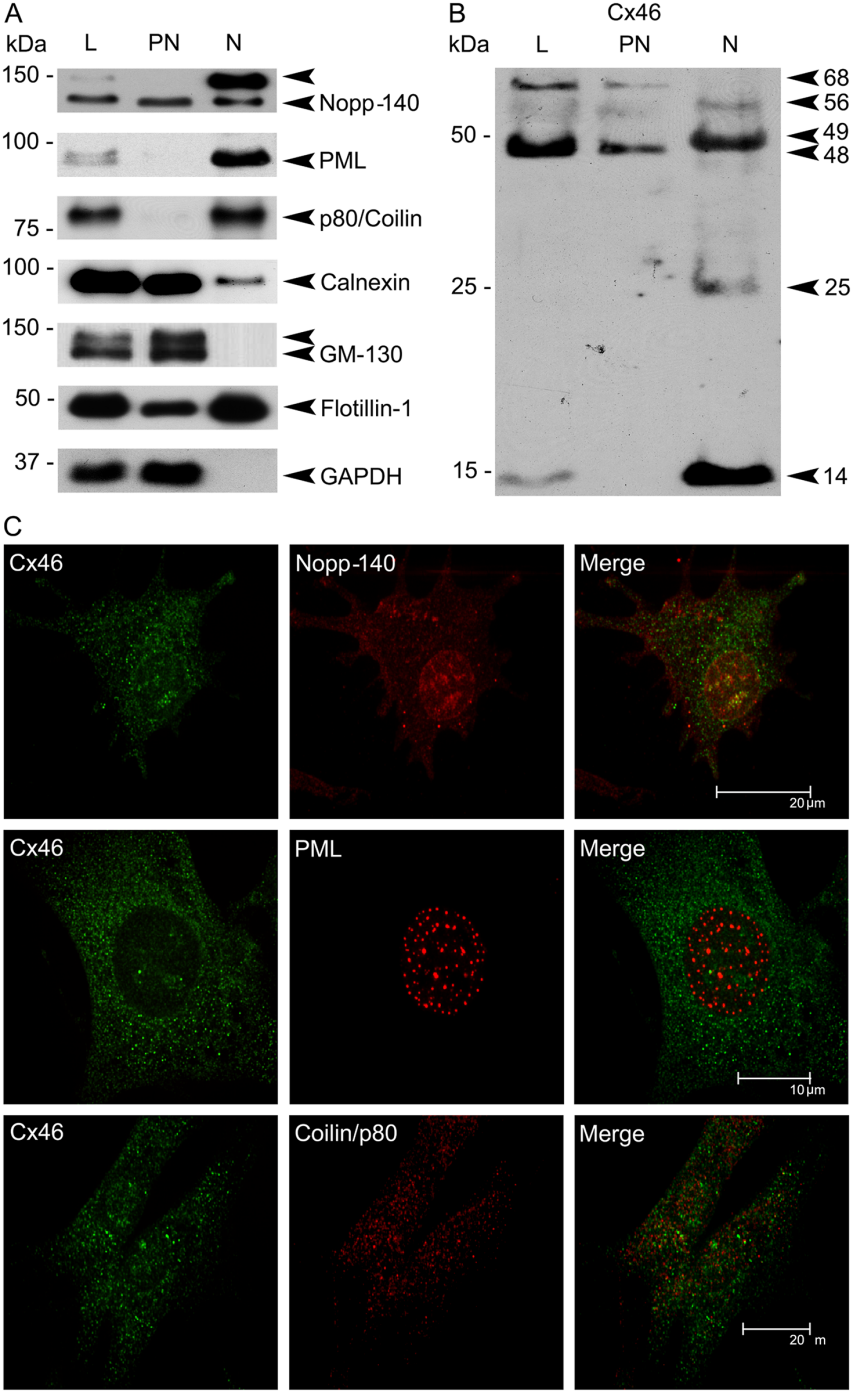
Characterization of nuclear Cx46 in TtT/GF FS cells. (A) and (B). Post-nuclear (PN) and nuclear (N) fractions were obtained from TtT/GF cell whole cell lysates (L). The fractions were subjected to SDS-PAGE and immunoblotting. (A) Characterization of the fractions: Membranes were probed with antibodies to nuclear structures (the nucleolus and Cajal bodies (Nopp-140), promyelocytic leukemia nuclear bodies (PML) and Cajal bodies (p80/coilin)), membrane domains: RER (calnexin), *cis*-Golgi (GM130), lipid rafts (flotillin-1)) and the cytosol (glyceraldehyde 3-phosphate dehydrogenase, GAPDH). (B) Membranes were incubated with an antibody against Cx46 (Alpha-Diagnostic). The 68, 56 and 48 kDa immunoreactive bands were recovered in the post-nuclear fraction whereas the 56, 49, 25 and 14 kDa bands were recovered in the nuclear faction. Western blots shown are representative of three independent experiments. (C) Confocal microscopy studies: TtT/GF cells were probed with Cx46 antibody and markers of nuclear structures: Nopp-140, PML and p80/coilin. Cells were visualized using confocal microscopy. Images shown correspond to a unique focal plane of 0.7 μm thickness. Micrographs are representative of three independent immunolabelling experiments. Cx46 and Nopp-140 partially co-localized in the nucleus. Nuclear Cx46 co-localized with neither PML nor p80/coilin.

The controls performed on *Cx46-/-* mouse tissue sections have been documented and reported elsewhere [42]. Controls realized on mink anterior pituitary paraffin sections with either the primary or secondary antibody alone showed no reaction products (Fig 1E). The FS cells set at the periphery of anterior pituitary follicles and their thin cytoplasmic processes between hormone secreting cells housed plentiful Cx46-positive dots (Fig 1E’ and 1E”). Cx46-positive dots occupied the endocrine cell perinuclear zone (Fig 1E”, asterisk). Significantly, the endocrine cell’s nucleolus was also Cx46-positive (Fig 1E”). A punctate immunofluorescence labelling was observed in TtT/GF cells probed for Cx46 being at times concentrated in sizeable perinuclear aggregates (Fig 1F). The TtT/GF cell cytoplasmic processes were weakly labelled and cell-to-cell contact areas were negative (Fig 1F). The TtT/GF cell nucleus was Cx46-positive (Fig 1F (insert)). This observation was confirmed by confocal microscopy (see Figs 2 and 3 for additional examples of Cx46 nuclear labelling).

**Fig 4:**
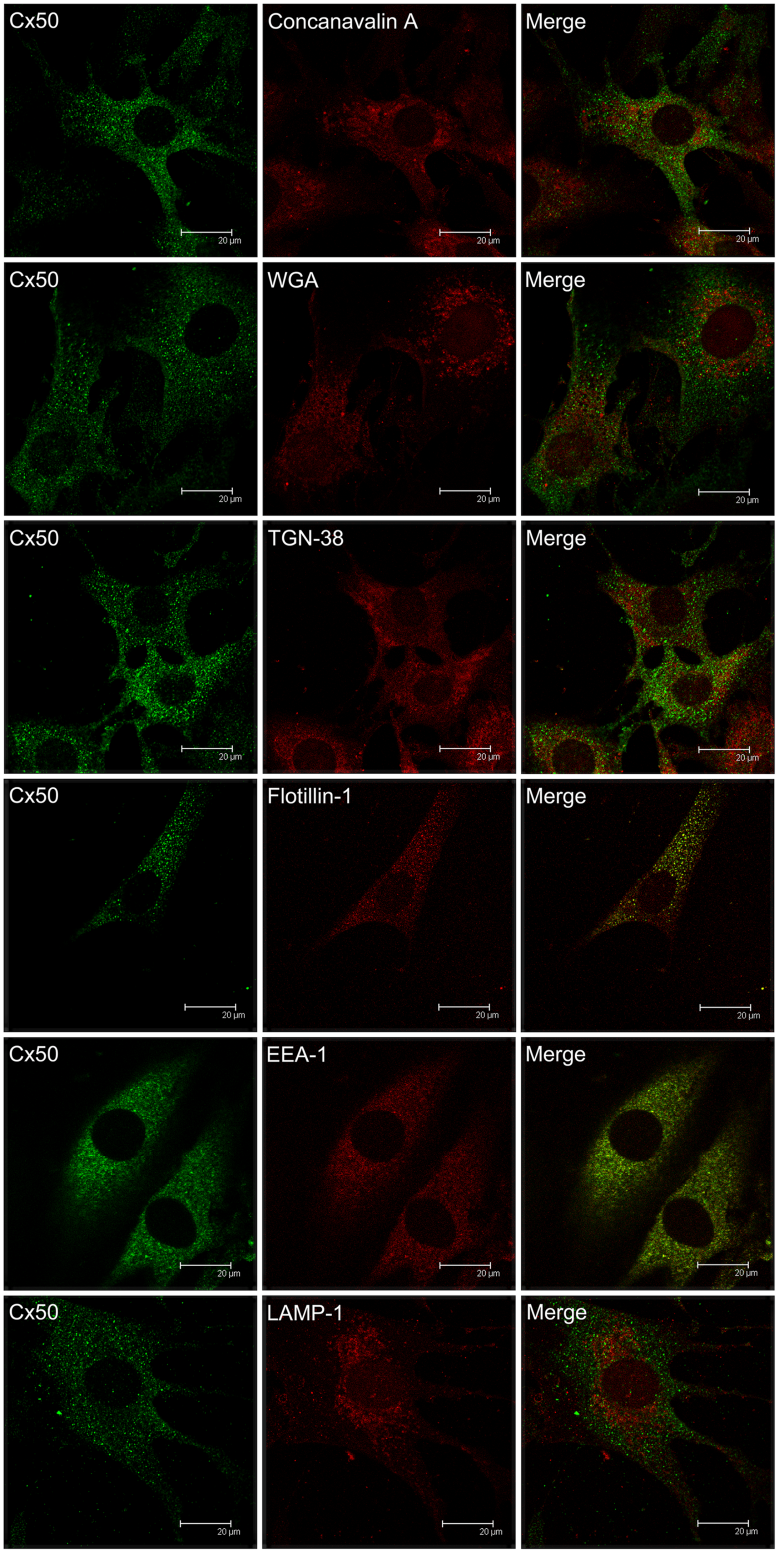
Confocal microscopy on the co-localization of Cx50 and cellular organelles in TtT/GF FS cells. TtT/GF cells were double-stained with Invitrogen Cx50 antibody and an organelle marker (antibody or probe). Preparations were visualized with a confocal microscope. Micrographs shown correspond to a unique focal plane of 0.7 μm thickness. Cx50 did not co-localize with the endoplasmic reticulum (concanavalin A), Golgi apparatus (WGA), *trans*-Golgi (TGN-38), early endosomes (EEA-1), or lysosomes (LAMP-1). A weak Cx50 co-localization with the lipid raft marker flotillin-1 was observed in the cytoplasm.

**Fig 5:**
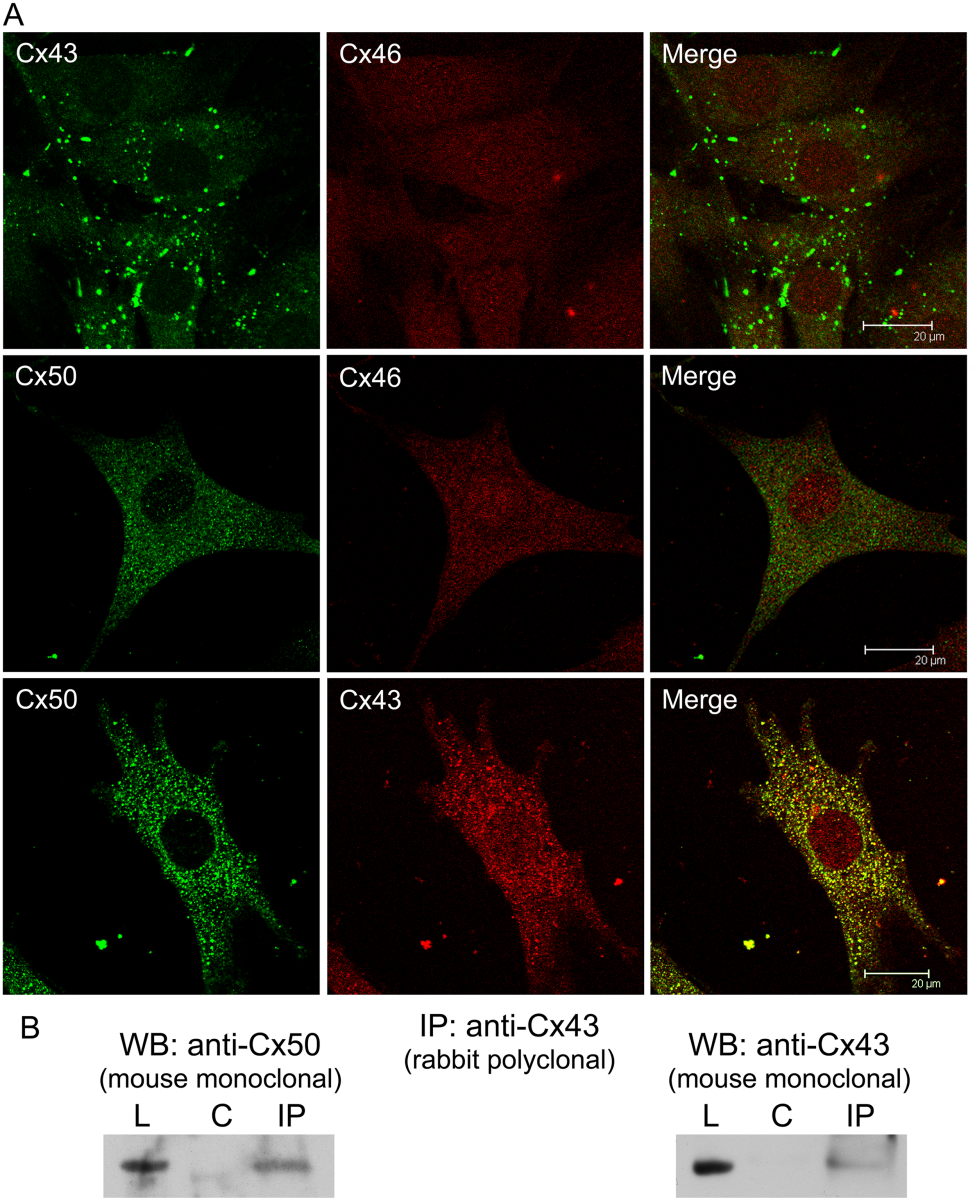
Cx43, Cx46 and Cx50 interaction in TtT/GF FS cells. (A) Confocal microscopy of TtT/GF cells labelled for Cx43, Cx46 and Cx50. Micrographs shown correspond to a unique focal plane of 0.7 μm thickness. Cx43-Cx46 co-localized neither in the cytoplasm nor in the nucleus. As well, no co-localization was apparent for Cx50-Cx46. Cx50 co-localized with Cx43 in the cytoplasm. (B) Co-immunoprecipitation studies. Pre-cleared TtT/GF cell lysates (L) were incubated either with buffer alone (control: C) or rabbit polyclonal anti-Cx43 (IP). Next, the mixtures were incubated with protein A Sepharose beads. Proteins attached to beads were subjected to SDS-PAGE followed by Western blotting (WB) with either mouse monoclonal anti-Cx50 or mouse monoclonal anti-Cx43. The figure shows representative membranes. Cx43 and Cx50 were both pulled down by Cx43 antibody.

The controls on *Cx50-/-* mouse tissue sections have been documented and reported elsewhere [42]. Controls on mink anterior pituitary sections with either the primary or the secondary antibody alone showed no reaction product (Fig 1G). Cx50 labelled FS cells at the periphery of the anterior pituitary follicles with their thin cytoplasmic processes bordering hormone secreting cells (Fig 1G’). The endocrine cells showed no reaction products (Fig 1G’, asterisk). The wall of blood vessels in the interstitium between follicles was Cx50-positive (Fig 1G’, bv). Cx50 immunofluorescence labelling of TtT/GF cells produced a punctate pattern throughout the cytoplasm and cytoplasmic processes (Fig 1H). Cx50 labelled the plasma membrane (Fig 1H) but not the cell nucleus (confirmed by confocal microscopy, Figs 4 and 5).

### Association of Cx46 with cytoplasmic organelles in TtT/GF FS cells

We assessed the association of Cx46 with individual organelles and domains within the cell using double labelling of Cx46 and specific organelle markers (Table 1). The isotype of the Cx46 antibodies available restricted us from double labelling cells with markers of identical isotype (IgG, made in rabbit).

Concanavalin A (RER marker) fluorescence pattern was recorded in the cytoplasm but not extending to the periphery of the cell. No co-localization of Cx46 with Concanavalin A was observed (Fig 2, Concanavalin A). Wheat germ agglutinin (WGA, Golgi apparatus) showed a vesicular-like staining pattern neighbouring the nucleus. Merged images of Cx46 and WGA labelling showed co-localization in few vesicles (Fig 2, WGA and S1 Fig for higher magnification images). The *trans*-Golgi network labelling with anti-TGN-38 was punctate and perinuclear. Merged TGN-38 and Cx46 images revealed no co-localization (Fig 2, TGN-38). Flotillin-1 (non-caveolar lipid rafts) exhibited a punctate pattern in the cytoplasm and the nucleus (Fig 2, Flotillin-1). Merged Cx46 and flotillin-1 images showed no co-localization in either the cytoplasm or the nucleus (Fig 2, Flotillin-1). The early endosomes were labelled with the EEA-1 antibody which displayed a cytoplasmic vesicular staining sometimes concentrated in the perinuclear area. A weak Cx46 and EEA-1 co-localization was observed in vesicles next to the nucleus (Fig 2, EEA-1 and S1 Fig for higher magnification images). Labelling of lysosomes with antibodies against the protein LAMP-1 identified rounded and elongated shaped formations in the perinuclear area. An intense Cx46 and LAMP-1 co-localization was apparent in the perinuclear area (Fig 2, LAMP-1 and S1 Fig for higher magnification images).

### Association of Cx46 with nuclear domains in TtT/GF FS cells

Post-nuclear (PN) and nuclear (N) fractions were obtained from whole TtT/GF cell lysates (L). The following nuclear domain markers (Table 1) were recovered chiefly in the nuclear fraction: Nopp-140 (nucleolus and Cajal bodies, particularly the hyper-phosphorylated form), PML (PML bodies) and p80/coilin (Cajal bodies) (Fig 3A). Nopp-140 and, in a lesser extent, p80/cofilin were also found in the post-nuclear. These proteins are known to shuttle between the nucleus and the cytoplasm [62;63]. The nuclear fraction was free of the *cis*-Golgi apparatus marker GM130 and cytosolic protein GAPDH but positive for calnexin and flotillin-1 (Fig 3A). The presence of calnexin in the nuclear fraction could be attributed to a potential contamination with RER membranes associated with the outer membrane of the nuclear envelop. The presence of flotillin-1 in the nucleus was confirmed by confocal microscopy (Fig 2, flotillin-1); however, no Cx46-flotillin-1 co-localization was apparent in the nucleus (Fig 2, flotillin-1). Cx46-positive bands exhibited a differential distribution in the post-nuclear and nuclear fractions (Fig 3B). The 68 kDa Cx46 was found principally in the post-nuclear fraction whereas the 56 kDa Cx46 was observed in both the post-nuclear and nuclear fractions. The 48 kDa Cx46 was recovered in the post-nuclear fraction, whereas the 49 kDa Cx46 was recovered in the nuclear fraction. The 25 and 14 kDa Cx46 were recovered solely in the nuclear fraction. Confocal microscopy shows nuclear Cx46 co-localization with Nopp-140 (Fig 3C and S1 Fig), but not with PML or p80/coilin (Fig 3C).

### Association of Cx50 with cytoplasmic organelles in TtT/GF FS cells

One of the Cx50 antibody isotypes used in our experiments was an IgM; this allowed double labelling Cx50 with a wider spectrum of organelle markers than when we used Cx46 antibodies (Table 1). Cx50 did not co-localize with the RER markers concanavalin-A (Fig 4) and calnexin (S2 Fig). Cx50 did not co-localize with the Golgi marker WGA and *trans*-Golgi TGN-38 (Fig 4), but a weak co-localization with GM-130 (*cis*-Golgi) was apparent in some perinuclear vesicles (S2 Fig). Cx50 partially co-localized with flotillin-1 (Fig 4) but not with caveolin-1 (S2 Fig). No Cx50 co-localization was apparent with either the endosome marker EEA-1 or the lysosome marker LAMP-1 (Fig 4).

### Interactions between Cx46, Cx50 and Cx43

Cx46 did not co-localize with either Cx43 or Cx50 in TtT/GF cells (Fig 5A). By contrast, Cx50 co-localized with Cx43 in the cytoplasm (Fig 5A and S3 Fig for a higher magnification image). The Cx43-Cx50 interaction was substantiated by immunoprecipitation studies. Cx43 polyclonal antibody pulled down both Cx43 and Cx50 (Fig 5B). Reversal immunoprecipitation attempts with the Cx50 antibody were unsuccessful.

Table 2 summarizes Cx46 and Cx50 interactions with subcellular domains and Cx43.

**Table 2:** Summary of the co-localization of Cx46 and Cx50 and different cellular markers.

Domain	Markers	Cx46	Cx50
Rough endoplasmic reticulum	Calnexin	N/A	no
Rough endoplasmic reticulum	Concanavalin A	no	no
*Cis*-Golgi	GM130	N/A	~ yes
Golgi and *Trans*-Golgi	WGA	~ yes	no
*Trans*-Golgi	TGN-38	no	no
Lipid rafts	Flotillin-1	no	~ yes
Nuclear flotillin-1	Flotillin-1	no	no
Caveolae	Caveolin-1	N/A	no
Gap junction	Cx43	no	yes
Gap junction	Cx46	-	no
Gap junction	Cx50	no	-
Early endosomes	EEA-1	~ yes	no
Lysosomes	LAMP-1	yes	no
Nucleolus-Cajal bodies	Nopp-140	yes	no
PML bodies	PML	no	no
Cajal bodies	Coilin/p80	no	no

### In vivo studies on the impact of physiological and pathological conditions that affect anterior pituitary hormone secretion on the expression, phosphorylation status and localization of Cx46 and Cx50 in the anterior pituitary

#### Lactation

**Fig 6:**
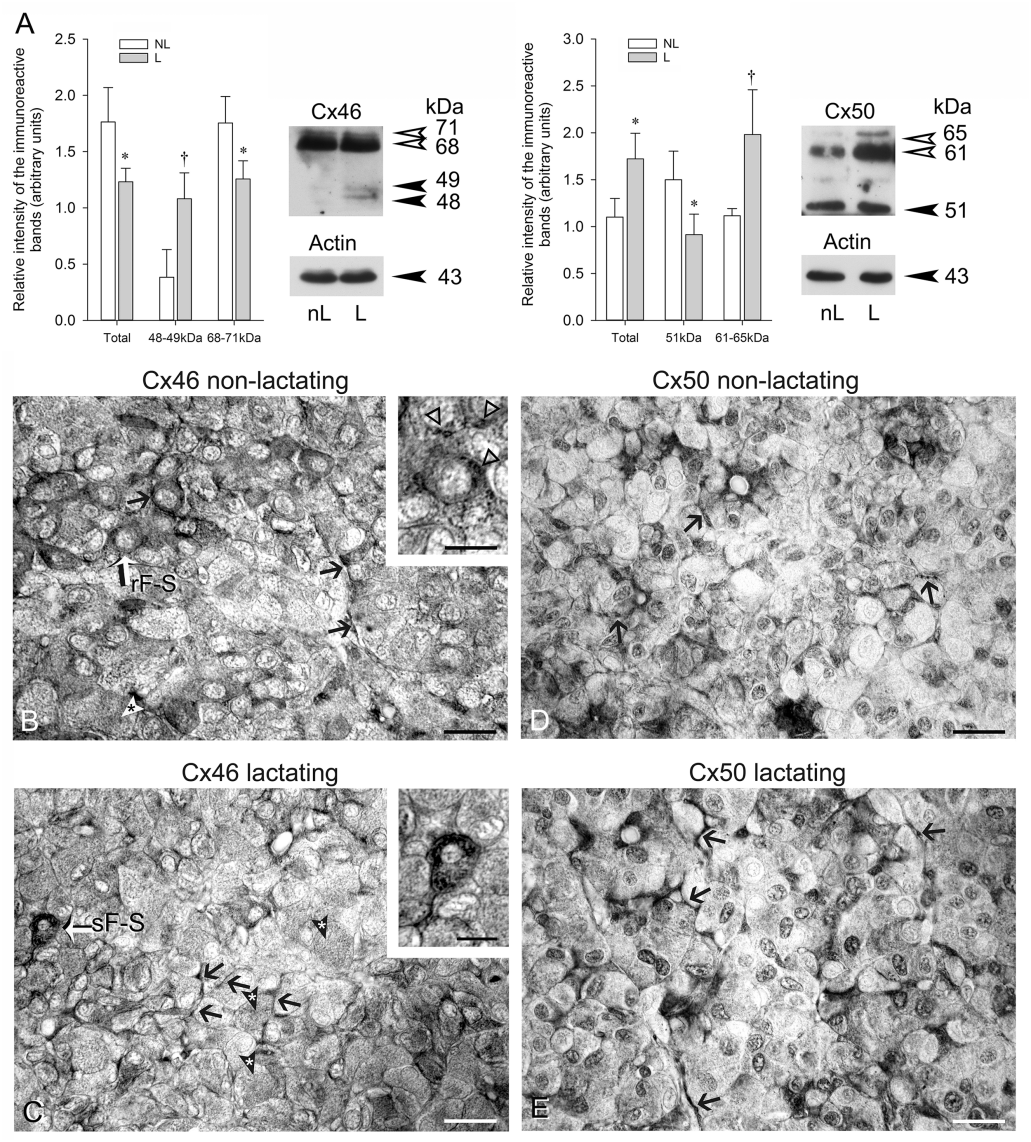
Cx46 and Cx50 in non-lactating and lactating female mink anterior hypophyses. (A) Western blot analyses of Cx46 and Cx50 in non-lactating (nL) and lactating (L) mink anterior pituitaries. Thirty μg total protein samples were subjected to SDS-PAGE and immunoblotting with Cx46 and Cx50 antibodies. Membranes were reprobed with monoclonal anti-actin and polyclonal anti-actin respectively. Representative Western blots are shown. Values are the mean ± SEM of three different animals per experimental condition. Intensity values were normalized to the corresponding actin value. Statistics (Student’s *t* test): Total Cx46 levels were lower in lactating than in non-lactating female mink (* P<0.05). The intensity of the 48–49 Cx46 band was higher († P<0.03) whereas that of the 68–71 kDa band was lower (* P<0.05) in lactating than non-lactating mink. Total Cx50 levels were higher in lactating than in non-lactating mink (* P<0.05). The 51kDa Cx50 band intensity was lower (* P<0.05) whereas that of the 61–65 kDa band was higher († P<0.03) in lactating than non-lactating mink. (B-E) Representative light micrographs of non-lactating (B and D) and lactating (C and E) mink anterior pituitary Bouin’s-fixed paraffin sections exposed to Cx46 (B and C) and Cx50 (D and E) antibodies respectively. A Cx46-positive round-shaped type 2 FS cell with a pale rounded nucleus is identified rFS in (B). In addition, open triangles point to Cx46-positive material in the perinuclear Golgi apparatus and lysosomes in three neighboring cells (insert). A type 1 stellate-shaped cell (identified sFS, white arrow) with its nucleus close to the center of the follicle is seen (C) containing plentiful Cx46-positive dots. (B and C): The many arrows indicate Cx46 labelling within the FS cell cytoplasmic processes bordering individual endocrine cells. The white (B) and the black (C) arrowheads with an asterisk point to endocrine cells containing minute Cx46-positive dots. In (D and E), the black arrows point to Cx50-positive FS cell cytoplasmic processes. In (D), a punctate pattern of Cx50 immunolabelling typical of gap junctions is apparent at the site of non-endocrine-endocrine cells contacts associated with plasma membranes. Bars: B, C, D and E: 50 μm; inserts in B and C: 25 μm.

Earlier we documented Cx43 level changes in the anterior pituitary [15] and reported that Cx43-positive, type 1 FS cells increase during periods associated with high Prl secretion such as lactation [8]. Fig 6A shows, in agreement with Fig 1A, that Cx46 appeared chiefly under phosphorylated forms (68–71 kDa) in mink anterior pituitaries. Total Cx46 levels significantly decreased during the lactation period; the decrease was mainly due to a reduction in the 68–71 kDa band intensity level (Fig 6A, Cx46). By contrast, 48–49 kDa Cx46 levels were significantly increased in lactating compared to non-lactating mink (Fig 6A, Cx46). The decrease in Cx46 phosphorylated forms combined with the augmenting 48–49 kDa suggest increased Cx46 dephosphorylation taking place during lactation. We found the 51 and 61–65 kDa Cx50 immunoreactive bands both apparent in female mink anterior pituitaries (Fig 6A, Cx50). In contrast to Cx46, total Cx50 was significantly higher during lactation (Fig 6A, Cx50); the increase was due to increased values of high molecular mass 61–65 kDa Cx50 phosphorylated forms. The levels of the 51 kDa Cx50 were lower in lactating than non-lactating mink (Fig 6A, Cx50).

The FS cells disposed at the periphery of anterior pituitary follicles and their delicate cytoplasmic processes rounding hormone secreting cells displayed intense Cx46-labelling in non-lactating mink anterior pituitary (Fig 6B). Fig 6C shows Cx46 labelling in lactating mink. The endocrine cells of both animal groups displayed minute Cx46-positive dots (Fig 6B and 6C). Fig 6D shows Cx50 immunoreactivity in FS cells and blood vessels in non-lactating mink anterior pituitary. FS cells and blood vessels in lactating mink anterior pituitaries exhibited intense Cx50 immunoreactivity (Fig 6E).

#### The male mink seasonal reproductive cycle

**Fig 7:**
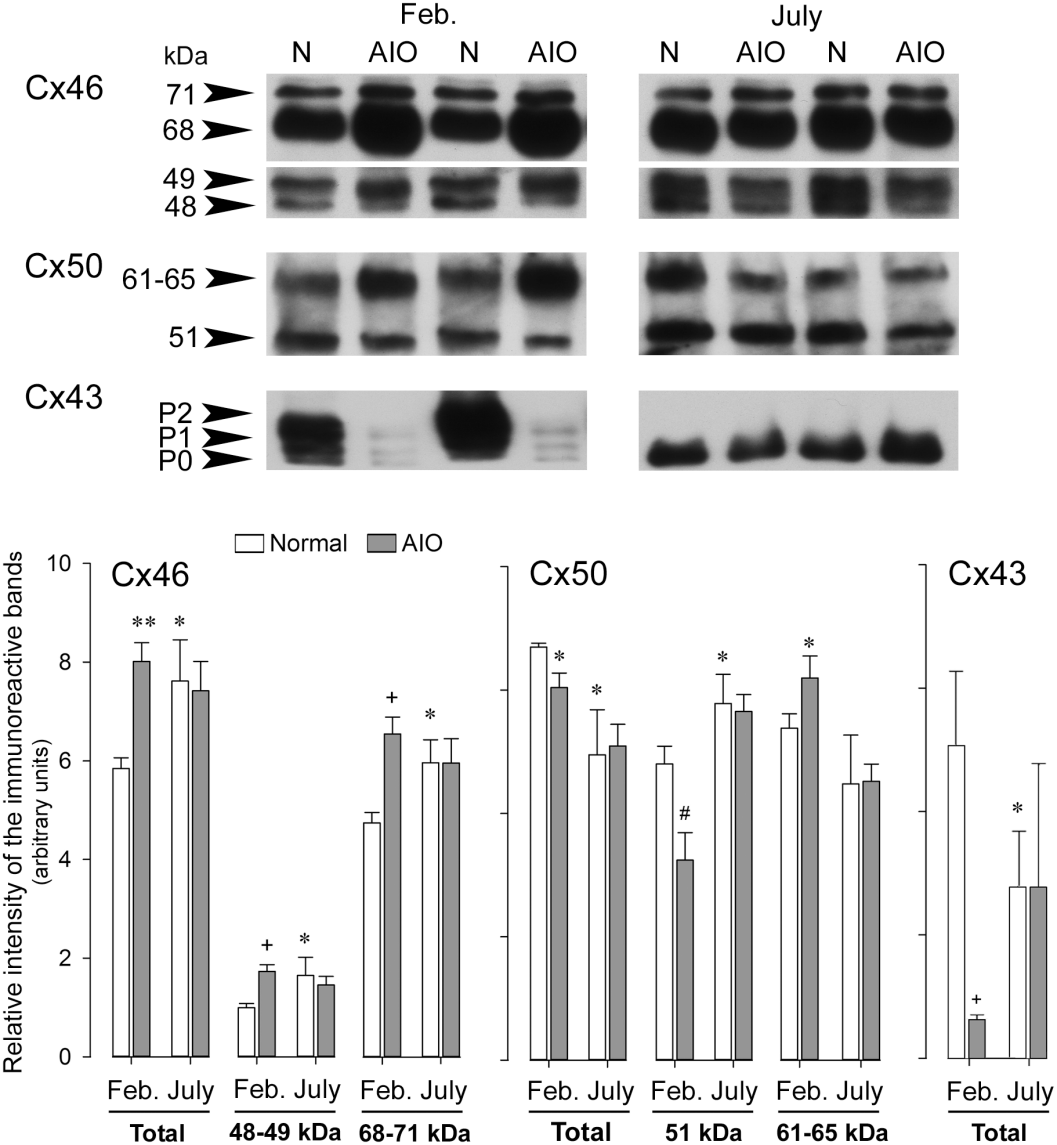
Cx46, Cx50 and Cx43 expression profiles in anterior pituitary during different periods of the annual seasonal reproductive cycle in normal male mink and during spontaneous autoimmune orchitis. Anterior pituitaries were harvested in normal (N) and orchitic (AIO) male mink in February (Feb., active spermatogenic phase of the annual reproductive cycle) and in July (inactive spermatogenic phase of the annual reproductive cycle). Twenty μg of total protein samples were loaded per well. After electrophoresis and transfer, membranes were probed with Cx46, Cx50 or Cx43 antibodies; next stripped and reprobed with monoclonal or polyclonal anti-actin. The figure shows representative Western blots. The bands were scanned and the intensity values were normalized to the corresponding actin band intensity value. The values shown are the mean ± SEM of three different animals per experimental condition. Statistics (Student’s *t* test): Normal male mink (comparison between open bars in February and July): Total, 48–49 and the 68–71 kDa Cx46 levels were higher in July than in February (* P<0.05). Total Cx50 was lower (* P<0.05) in July than in February whereas 51 kDa Cx50 was higher (* P<0.05) in July than in February. Total (P0+P1+P2) Cx43 levels were lower in July than in February (* P<0.05). AIO mink (comparison between open bars and grey bars within the same month). February: Total Cx46 (** P<0.005), 48–49 kDa Cx46 (+ P<0.01) and 68–71 kDa Cx46 (+ P<0.01) levels were all increased in orchitic compared to normal mink. Total Cx50 (* P<0.05) and 51 kDa Cx50 (# P<0.02) levels were lower in orchitic than normal mink, whereas 61–65 kDa was higher (* P<0.05) in mink with AIO than in normal mink. Total (P0+P1+P2) Cx43 levels decreased in AIO mink than in normal mink (* P<0.05). No differences were registered for any of the Cxs studied between orchitic and normal mink in July.

We have established the male mink as a valuable model for the study of Cx43 involvement in reproduction [64–66]. The active spermatogenic phase (December to March in the Northern hemisphere) is characterized by elevated serum gonadotropins but lowered Prl [49;50]. Here, we assessed the changes in Cx46, Cx50 and Cx43 expression profiles in male mink anterior pituitary during the natural hormonal changes that take place during distinct time periods of the annual seasonal reproductive cycle. In normal mink anterior pituitaries, the levels of total Cx46 and individual Cx46 forms significantly augmented during testicular regression (July) compared to the active spermatogenic phase (Feb.) of the annual seasonal reproductive cycle (Fig 7 Cx46, Normal: open bars). Conversely, the total Cx50 levels dropped in July (Fig 7 Cx50, Normal: open bars). However, the levels of 51kDa Cx50 significantly increased, whereas Cx50 phosphorylation (61–65 kDa band) tended to decrease in July (Fig 7 Cx50, Normal: open bars). Cx43 antibody recognized three immunoreactive bands in normal mink anterior pituitaries harvested in February: P0: ~ 40 kDa, P1: ~ 42–44 kDa and P2: ~ 46-48kDa. Total (P0+P1+P2) Cx43 levels were lower in July than in February (Fig 7 Cx43, Normal: open bars). The P2 band which includes Cx43 phosphorylated forms was no longer apparent in July in contrast to February (Fig 7 Cx43, Normal: open bars).

#### Spontaneous autoimmune orchitis

We have shown that spontaneous AIO disrupts the normal hormonal profile in mink particularly during the active spermatogenic phase (February) [49;50]. Here, we show that AIO significantly altered Cx46, Cx50 and Cx43 levels in the anterior pituitary in February but not in July. The levels of total, 48–49 and 68–71 kDa Cx46 in mink with AIO compared to normal mink increased in February (Fig 7, Cx46) whereas there were no significant differences in July (Fig 7, Cx46). Total Cx50 levels significantly decreased in AIO compared to normal mink in February (Fig 7, Cx50). Fifty-one kDa Cx50 levels decreased by contrast to 61 kDa Cx50 levels which increased in AIO mink compared to normal mink in February. These changes suggest increased Cx50 phosphorylation and degradation in orchitic animals. The levels of total, 51and 61–65 kDa Cx50 in AIO and normal mink in July were similar (Fig 7, Cx50). Total Cx43 levels decreased in the anterior pituitary of mink with AIO compared to normal in February (Fig 7, Cx43). No changes were registered in July (Fig 7, Cx43).

#### The leptin-deficient (*ob/ob*) and leptin receptor-deficient (*db/db*) mice

**Fig 8:**
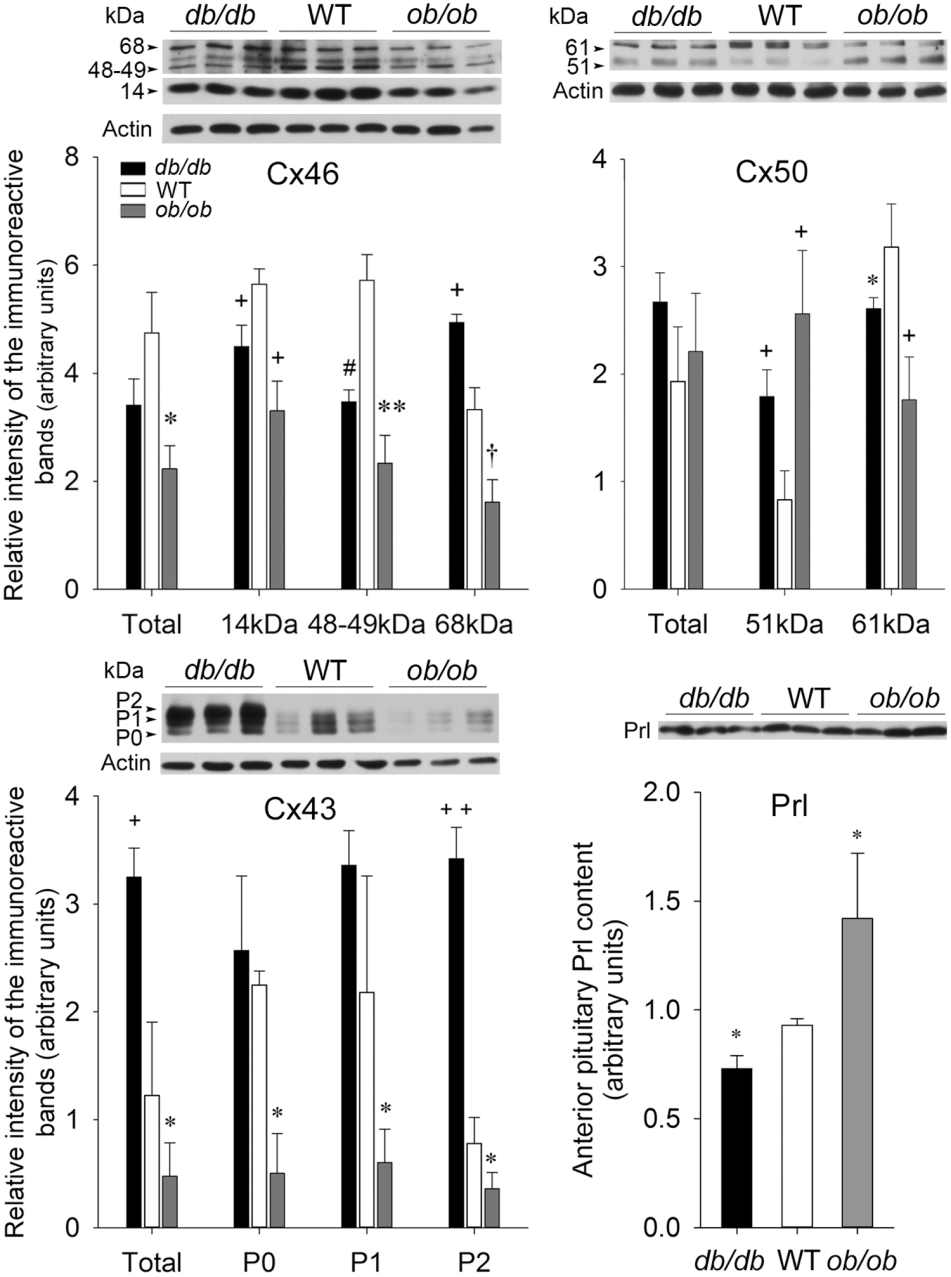
Cx46, Cx50 and Cx43 expression in wild type mice and in leptin receptor- (*db/db*) and leptin- (*ob/ob*) deficient male mice. Wild type (WT), leptin receptor- (*db/db*) and leptin- (*ob/ob*) deficient male mouse anterior pituitaries were harvested. Thirty μg of total protein samples were loaded per well. After electrophoretic migration, proteins were electrotransferred onto nitrocellulose membranes and probed with either Cx46, Cx50, Cx43 or Prl antibodies. Membranes were reprobed with monoclonal or polyclonal anti-actin. Bands were scanned and their intensity quantified. The values obtained were normalized to the corresponding actin band intensity value. The figure shows representative Western blots. The values are the mean ± SEM of three different animals per experimental condition. Statistics (Student’s *t* test): Cx46: Total Cx46 levels were not significantly different in *db/db* and in WT mice. However, the intensities of the 14 and 48–49 kDa Cx46 immunoreactive bands were lower in *db/db* than in WT mice (+ P<0.01 and # P<0.02 respectively) whereas that of 68 kDa was stronger *db/db* than WT mice (+ P<0.01). Total, 14, 48–49 and 68 kDa Cx46 levels were significantly decreased (* P<0.05, + P<0.01, ** P<0.005 and † P<0.03 respectively) in *ob/ob* compared to WT mice. Cx50: Total Cx50 levels showed little change in *db/db* and *ob/ob* mice compared to WT. The intensity of the 51 kDa Cx50 immunoreactive band was more intense in *db/db* and *ob/ob* mice compared to WT mice (+ P<0.01). By contrast, the intensity of the 61 kDa-immunoreactive band was weaker in *db/db* and *ob/ob* mice compared to WT mice (* P<0.05 and +P<0.01 respectively). Cx43: Cx43 total levels (P0+P1+P2) increased in *db/db* compared to WT mice (+ P<0.01) due to the increase in the phosphorylated P2 band (++ P<0.001). P0 and P1 Cx43 showed no significant differences in *db/db* and WT mice. However, all Cx43 isoforms decreased in *ob/ob* compared to WT mice (* P<0.05). Prl: Anterior pituitary Prl content decreased (* P<0.05) in *db/db* but increased (* P<0.05) in *ob/ob* mice compared to the WT counterparts.

We used the leptin-deficient *ob/ob* and the leptin receptor-deficient *db/db* mice to assess the effect of an altered anterior pituitary functioning on Cx46, Cx50 and Cx43 expression and phosphorylation. The impact of leptin on anterior pituitary hormone secretion through its action on the anterior pituitary gland [51;55] and on FS cell gap junctions [22] is well documented. Moreover, both mice are obese and diabetic, two pathological conditions which have been reported to modify Cx levels in a wide range of tissues in the body [46;54]. Total Cx46 levels were not significantly different in *db/db* and WT anterior pituitaries (Fig 8, Cx46, *db/db*). However, 14 and 48–49 kDa Cx46 levels significantly diminished whereas 68 kDa Cx46 increased in *db/db* mice compared to the WT counterparts (Fig 8, Cx46, *db/db*). By contrast, 14, 48–49 and 68–71 kDa Cx46 levels all significantly decreased in *ob/ob* mice compared to WT (Fig 8, Cx46, *ob/ob*). Total Cx50 levels changed little in *db/db* and *ob/ob* mice compared to WT mice due to an increase in 51 kDa Cx50 levels and a concomitant decrease in Cx50 phosphorylated form levels (61–65 kDa) in *db/db* and *ob/ob* mice compared to WT counterparts (Fig 8, Cx50). The total Cx43 levels (P0+P1+P2), particularly the phosphorylated forms P1 and P2, were significantly increased in *db/db* compared to WT mice (Fig 8, Cx43, *db/db*). On the contrary, all Cx43 isoforms significantly decreased in *ob/ob* compared to WT counterparts (Fig 8, Cx43, *ob/ob*). The anterior pituitary Prl content significantly decreased in *db/db* mice whereas it increased in *ob/ob* mice in comparison to WT (Fig 8, Prl).

### In vitro studies: Effect of bFGF treatment on Cx46 and Cx50 levels in TtT/GF cells

**Fig 9:**
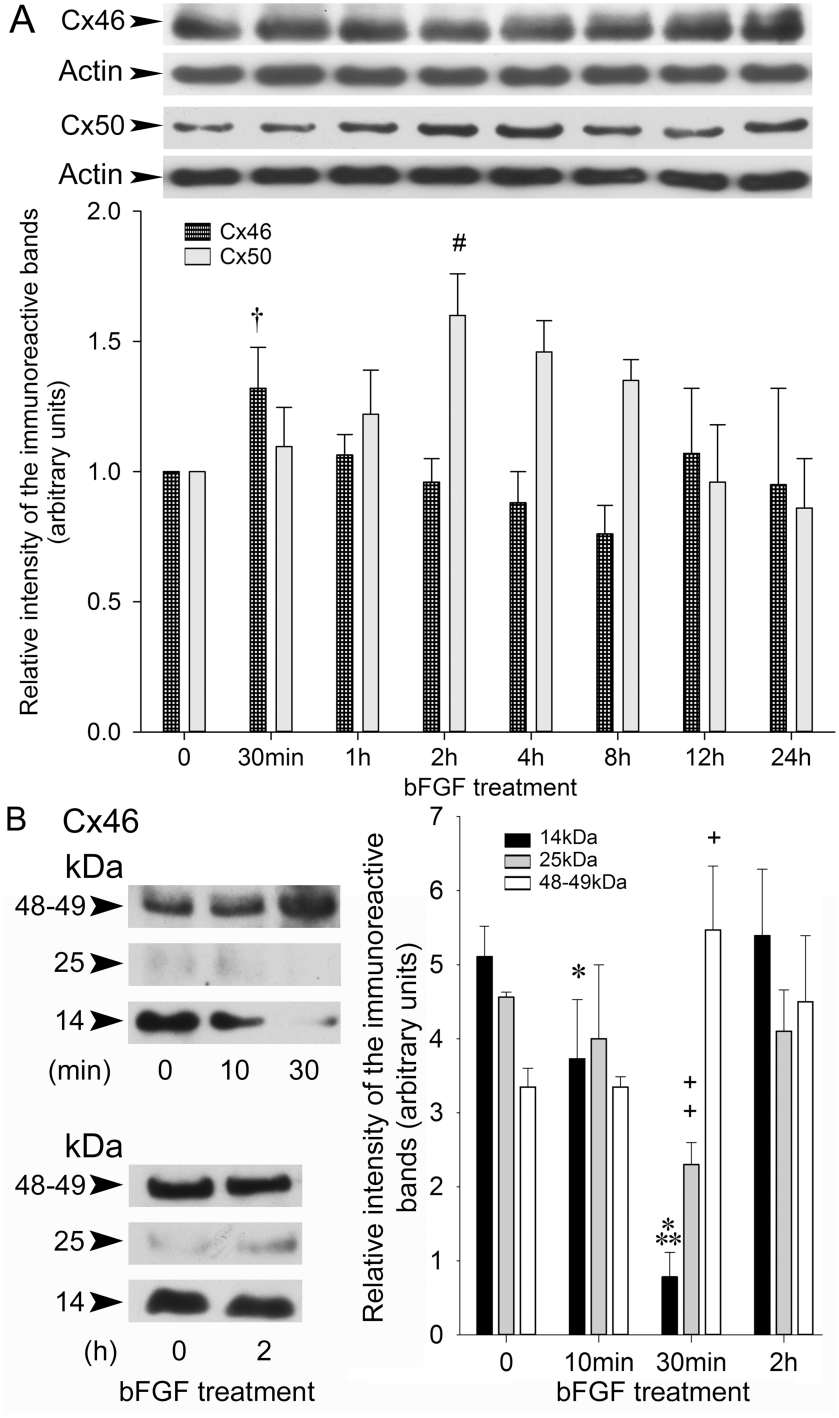
Effects of bFGF on Cx46 and Cx50 in TtT/GF FS cells. Serum starved TtT/GF cells were incubated with 15 ng/ml bFGF for increasing periods of time. Whole cell lysates (15 μg) were subjected to SDS-PAGE followed by electrotransfer. Membranes were incubated with Cx46 or Cx50 antibodies. The membranes were stripped and reprobed with mouse monoclonal anti-actin and polyclonal anti-actin respectively. (A) Representative Western blots are shown. The bands were scanned and their intensity values were normalized first, to the corresponding actin band intensity value, next to the respective control value (time = 0). Values shown are the mean ± SEM of three independent experiments. Statistics (ANOVA followed by THSDT). Forty eight-49 kDa Cx46 was transiently and significantly increased by 30 min in the presence of bFGF († P<0.03). Cx50 increased after a 2 h incubation period with bFGF (# P<0.02 2 h vs 0 h), then slowly receded to control values by 24h in the presence of bFGF. (B) Following short-term incubation with bFGF, the 14, 25 and 48–49 kDa Cx46 immunoreactive band intensities were quantified. Values shown are the mean ± SEM of three independent experiments. Statistics (ANOVA followed by THSDT): Short-term incubation with bFGF decreased the intensity of the14 kDa immunoreactive band (black bars) by 10 min (* P<0.05 10 min vs 0 min) and by 30 min (*** P<0.0005 30 min vs 10 min). Short-term treatment with bFGF decreased the intensity of the 25 kDa immunoractive band (grey bars) by 30 min (++ P<0.001 30 min vs 0 min). Forty eight-49 kDa Cx46 immunoreactive band intensity (open bars) was increased at 30 min (+ P<0.01, 30 min vs 10 min).

Earlier, we showed that the growth factor bFGF alters Cx43 expression and phosphorylation in TtT/GF cells [12]. Here, we assessed the action of bFGF on the expression of Cx46 and Cx50 in these cells. A short-term incubation of TtT/GF cells with bFGF rapidly (30 min) but transiently increased 48–49 kDa Cx46 levels (Fig 9A) whereas a longer exposure to the growth factor (2 h) transiently increased Cx50 levels (Fig 9A). To elucidate the mechanisms of the increase in 48–49 kDa Cx46 by a short-term incubation with bFGF, we measured whether bFGF affected the 14 and 25 kDa Cx46 nuclear forms. Incubating the cells 10–30 min with bFGF significantly and transiently decreased 14 kDa (black bars) and 25 kDa Cx46 (grey bars). By 30 min, 48–49 kDa Cx46 (white bars) significantly increased (Fig 9B). Control values for each band were recovered by 2 h in the presence of bFGF (Fig 9B).

## Discussion

Here, we show that Cx46 and Cx50 gap junction proteins belonging to the α-family are expressed in the anterior pituitary and, particularly in FS cells. The data provide evidence of significant differences in the cellular expression and subcellular distribution of these two Cx species as well as in their individual responses to physiological and pathological challenges. The results are indicative of distinctive actions of each Cx in the anterior pituitary and in FS cells. The data suggest that Cx46 is involved in the control of cell growth and proliferation and that Cx50, together with Cx43, contributes to FS cell coupling.

### Connexin 46

The Western blot analyses identified several Cx46 immunoreactive bands in the mouse anterior pituitary and TtT/GF cells. Most Cx46 immunoreactive bands in mink anterior pituitary homogenates were of a molecular mass higher than 48-49kDa confirming our earlier reports in testis [42]. The high weighted Cx46 immunoreactive bands are phosphorylated forms chiefly associated with the crude membrane fraction [42]. In addition, we detected low molecular mass Cx46 immunoreactive bands (14 kDa and 25 kDa) which corresponded to C-terminal fragments of the protein since antibodies used here were raised against the Cx46 C-terminal domain.

We localized Cx46 to endocrine cells and FS cells of the anterior pituitary as well as to cultured TtT/GF cells. Cx46 was chiefly concentrated in perinuclear vesicles and within the nucleus but only rarely associated with the cell membrane. These observations confirm our earlier reports in Sertoli cells and spermatocytes [42] and agree with published findings of Cx46 immunoreactivity in the perinuclear region of osteoblasts [32], lung adenomas [67], bone tissue [34], and Cx46-transfected lens epithelial cells [68]. In general, Cxs oligomerize in the Golgi apparatus before being delivered to the cell membrane by vesicular transport. Retention of monomeric Cx46 in the *trans*-Golgi network was reported in rat osteoblastic cells [32]; however, in our hands, Cx46 and the *trans*-Golgi network marker TGN-38 did not co-localize in TtT/GF cells. Nevertheless, our finding of Cx46 co-localized with the Golgi apparatus marker WGA but not with TGN-38 may indicate that some Cx46 molecules localize in Golgi apparatus domains different from the TGN. Cx46 and EEA-1-positive vesicles partially co-localized in the perinuclear region. This observation together with our finding of scarce Cx46 at the plasma membrane during “in vivo” and “in vitro” experiments suggests that, if Cx46 was sent to the membrane after its synthesis, the protein would be rapidly endocytosed. The Cx46-containing endosomes may then fuse with primary lysosomes for Cx46 degradation or processing. In agreement with this notion, most cytoplasmic Cx46 localized to lysosomes. Alternatively, Cx46 molecules may be distributed to lysosomes pinched off Golgi cisternae, thus bypassing the cell membrane transport. This notwithstanding, regardless of the mechanisms causing Cx46 to reside in lysosomes, the physiological significance of lysosomal Cx46 is elusive. Clues may emerge from our finding of Cx46 in the nucleus.

The presence of Cx46 in the cell nucleus was confirmed by subcellular fractionation and confocal microscopy in TtT/GF cells. The 14, 25 and 49 kDa Cx46 were exclusively present in the nuclear fraction. The 14 and 25 kDa fragments may result from the cleavage of cytoplasmic facing domains of lysosomal membrane-bound Cx46 i.e., the intracellular loop, and N- and C-terminal regions. Cleavage of the Cx46 intracellular loop [69;70] and C-terminal region [69;71;72] has been documented in lens fibers. As discussed above, intracellular loop fragments cannot be detected in the present studies because the antibodies used here only detect the Cx46 breakdown products containing the C-terminal of the protein. This entails that the 14 and 25 kDa Cx46 are C-terminal fragments of full length Cx46. These small fragments can be imported into the nucleus. Alternatively, the exclusively nuclear 14 and 25 kDa Cx46 could be generated within the nucleus itself and result from the cleavage of the full length protein catalyzed by nuclear proteases.

Cx46 is an intrinsic membrane protein. The localization of full length Cx46 molecules in the nucleus is intriguing since the process would require beforehand dissociation of the protein from the membrane where it had been inserted during its synthesis in the ER. Under our experimental conditions, the nuclear fraction shows a small contamination with ER which could be attributed to the continuity of the ER membrane with the nuclear envelop outer membrane. However, the full length Cx46 we recovered in the nuclear fraction is unlikely associated with ER. Cx46 and the ER marker Concanavalin A did not co-localize. Moreover, a possible contamination with the ER cannot account for our finding of only the 49 without the 48 kDa Cx46 in the nuclear fraction. Some cytokine and growth factor receptors in the cell membrane undergo endocytosis and become inserted into the nuclear envelop inner membrane (reviewed in [73]). However, under our experimental conditions, Cx46 labelling was intra-nuclear, not associated with the nuclear envelop. Interestingly, full length Cx43 [74;75] and Cx30 [76] have been reported in nuclei of transfected cells. Moreover, the full-length FGF receptor is imported into the nucleus via an importin-β-dependent mechanism after its binding to bFGF (reviewed by [73]). The nuclear import of 49 kDa Cx46 molecules could use a similar mechanism. After endocytosis, 49 kDa Cx46 could be Areleased@ from the lysosome membrane then, imported to the nucleus. The import of the 49 but not the 48 kDa Cx46 may conceivably require a “signal” present in 49 but absent in 48 kDa Cx46. This “signal” could account for the molecular weight differences. Alternatively, a full length nuclear 49 kDa Cx46 could be generated within the nucleus, since we did not detect the 49 kDa form in the post-nuclear fraction. The finding of functional nuclear ribosomal subunits is suggestive of a nuclear protein synthesis particularly in the nucleolus [77].

The presence within the nucleus of domains enriched in factors associated with particular activities is typical of the nuclear organization. Our confocal microscopy studies demonstrated the co-localization of Cx46 with Nopp-140. Nopp-140 is a phosphoprotein shuttling between the cytoplasm and the nucleus where it associates preferentially with the nucleolus (see review on Nopp-140 [63]). In the nucleolus, Nopp-140 localizes to the fibrillar center (FC) and dense fibrillar center (DFC) never to the granular compartment (GC). The FC and DFC are the nucleolar compartments where rRNA synthesis and processing take place, whereas GC is the site for pre-ribosome assembly. Nopp-140 interacts with two major classes of small nucleolar ribonucleoproteins (sno-RNPs) which catalyse rRNA modifications, and with RNA polymerase I. All these actions support the view of Nopp-140 engagement in nucleolus functioning particularly in rRNA biogenesis. Nopp-140 also localizes to Cajal bodies where it interacts with snoRNPs and with p80/coilin (reviewed in [63]. Since we did not detect co-localization of Cx46 with p80/coilin, Nopp-140-Cx46 interaction is likely taking place in the nucleolus and suggests involvement of Cx46 or its cooperation in Nopp-140-mediated activities such as the synthesis and modification of rRNA.

Cxs have been recognized as sensors of the cell cycle progression influencing cell growth independently of their cell-to-cell coupling activities. Specifically, Cx46 expression either inhibits or promotes growth depending on the experimental design and cell type [78]. Our results on the effect of the growth factor bFGF on nuclear Cx46 fragments support the view that Cx46 may contribute to the regulation of cell growth and proliferation. bFGF stimulates TtT/GF cell proliferation [79]. The rRNA synthesis and post-transcriptional modifications are key steps in cellular growth and proliferation. We found that an early response of TtT/GF cells to bFGF treatment was a transient reduction in 14 kDa and 25 kDa Cx46 nuclear fragments. Despite the unresolved identity of “Cx46 molecules” associated with Nopp-140, our observation of the nuclear Cx46 fragment levels being altered by bFGF treatment is suggestive of their participation in bFGF-induced physiological changes in TtT/GF cells.

To further determine the link between Cx46 and anterior pituitary function, we assessed the impact of physiological and pathological conditions that alter anterior pituitary endocrine function on Cx46. The data show that physiological conditions in the female characterized by high serum Prl levels such as lactation, decrease anterior pituitary Cx46 expression and phosphorylation. In the male however, Cx46 levels were reduced when serum gonadotropins seasonally increase and Prl decreases. AIO, a pathological condition characterized by decreased gonadotropin serum levels during the active phase of the annual reproductive cycle in mink [49;50], was accompanied by augmented anterior pituitary Cx46 levels. An imbalance in anterior pituitary hormone serum levels characterizes the leptin-deficient *ob/ob* and the leptin receptor-deficient *db/db* mice [51;53;80;81]. Leptin is known to stimulate LH, FSH, GH and Prl secretion by acting on the hypothalamus and the anterior pituitary [51;82;83]. Under our experimental conditions, the anterior pituitary Prl content was decreased in *db/db* mice but increased in *ob/ob* mice compared to WT mice. We found that leptin deficiency caused a reduction of all Cx46 forms in the anterior pituitary, whereas leptin receptor deficiency, which is characterized by high levels of leptin, resulted in decreased 14 and 48–49 kDa Cx46 and enhanced Cx46 phosphorylation. Together, our results show that normal and pathological conditions affecting pituitary endocrine function correlate with an altered Cx46 turnover in the gland.

The present data identified significant differences in the behavior of Cx46 and Cx43 in the anterior pituitary. Firstly, Cx46 and Cx43 exhibited no physical interaction, they co-localized neither in the cytoplasm nor in the nucleus in TtT/GF cells. Under basal conditions, Cx46 was associated with lysosomes not with the cell membrane, whereas Cx43 localizes to the cell membrane and cytoplasmic vesicles which are neither ER, nor Golgi cisternae nor lysosomes as we reported earlier [23]. Secondly, a short-term treatment with bFGF increased full length Cx46 levels (this paper) without modifying full length Cx43 levels [12]. Conversely, a bFGF long-term treatment transiently increases Cx43 synthesis and cell-to-cell communication [12] without modifying Cx46 full length levels (this paper). Thirdly, Cx46 and Cx43 exhibit opposite behaviors in the anterior pituitary of lactating female mink, in normal and orchitic male mink as well as in mice with *ob* or *db* mutation. Our data concur with our earlier report of an opposite Cx43 and Cx46 behavior in the testis [42].

The present data show that the regulation of Cx46 expression is sex-, cell- and tissue-dependent and that it greatly differs from that of Cx43. In addition, our results suggest that Cx46 is associated with cellular activities such as cell growth or proliferation in the anterior pituitary.

### Connexin 50

Immunoblotting detected distinct Cx50 immunoreactive bands in TtT/GF cells as well as in mouse and mink anterior pituitaries that are similar to those we described in testis [42]. Earlier, we reported that high molecular mass Cx50 immunoreactive bands are phosphorylated forms of the protein [42]. Here, we extended this observation by showing that most Cx50 phosphorylated molecules were recovered in the membrane crude fraction. Anterior pituitary Cx50 levels and phosphorylation were altered during lactation, the annual reproductive cycle, spontaneous AIO and following *ob* or *db* mutations. We have reported changes in Cx50 phosphorylation status during development, the annual reproductive cycle and spontaneous orchitis in testis [42]. Several kinases phosphorylate Cx50 with differential impact on Cx50 channel activity [69;84–86].

The present study identified similarities in the expression, localization and action of Cx50 and Cx43 while documenting significant differences between Cx50 and Cx46 in the anterior pituitary. Like Cx43 [15], Cx50 localized in the walls of anterior pituitary blood vessels whereas Cx46 did not. Unlike Cx46, Cx50 did not localize to endocrine cells. In FS cells *in situ* as well as in cultured TtT/GF cells, Cx50 was present in tiny dots distributed throughout the cytoplasm and cytoplasmic processes, less frequently, at the cell membrane. Contrarily, cytoplasmic Cx46 was chiefly perinuclear. Cytoplasmic Cx50 co-localized with Cx43 not with Cx46. Endogenous Cx50 and Cx43 in TtT/GF cell lysates interacted with each other suggesting assembly of heteromeric connexons in these cells. In contrast to Cx43 and Cx46, Cx50 was excluded from the cell nucleus.

Our data also evidenced that Cx50 and Cx46 responses to various challenges were opposite while Cx50 and Cx43 responses tended to be similar. In occurrence, Cx50 and Cx43 levels were both elevated during the active phase of the normal reproductive cycle and both decreased in AIO mink during the same period. Both Cx50 and Cx43 phosphorylation were higher in *db/db* than in *ob/ob* mice. Cx50 (this paper) and Cx43 [12;15] expression increased in the anterior pituitary during lactation and during long-term treatment of TtT/GF cells with bFGF.

These observations together with Cx50 and Cx43 co-localization and physical interaction prompt us to suggest that Cx50 collaborates with Cx43-mediated activities in FS cells.

## Conclusion

Here we showed for the first time that, besides Cx43, two additional α-Cxs, Cx46 and Cx50, are expressed in the anterior pituitary, specifically, in FS cells and in the FS cell line TtT/GF. Our subcellular distribution, biochemical and physiological studies favor the notion that both Cxs play different roles in the anterior pituitary. Further studies will elucidate the specific roles of each of these Cxs in the anterior pituitary.

## Supporting information

S1 Fig Confocal microscopy on Cx46 co-localization with WGA, EEA-1, LAMP-1 and Nopp-140 in TtT/GF FS cells. (TIF)

S2 Fig Confocal microscopy on the co-localization of Cx50 and cellular organelles in TtT/GF FS cells. (TIF)

S3 Fig Confocal microscopy on the co-localization of Cx50 and Cx43 in TtT/GF FS cells. (TIF)
