# Supplementary figures and images for: Distinctive actions of connexin 46 and connexin 50 in anterior pituitary folliculostellate cells
Source: PLoS One. 2017 Jul 31;12(7):e0182495. doi: 10.1371/journal.pone.0182495 (PMC5536325; doi:10.1371/journal.pone.0182495)

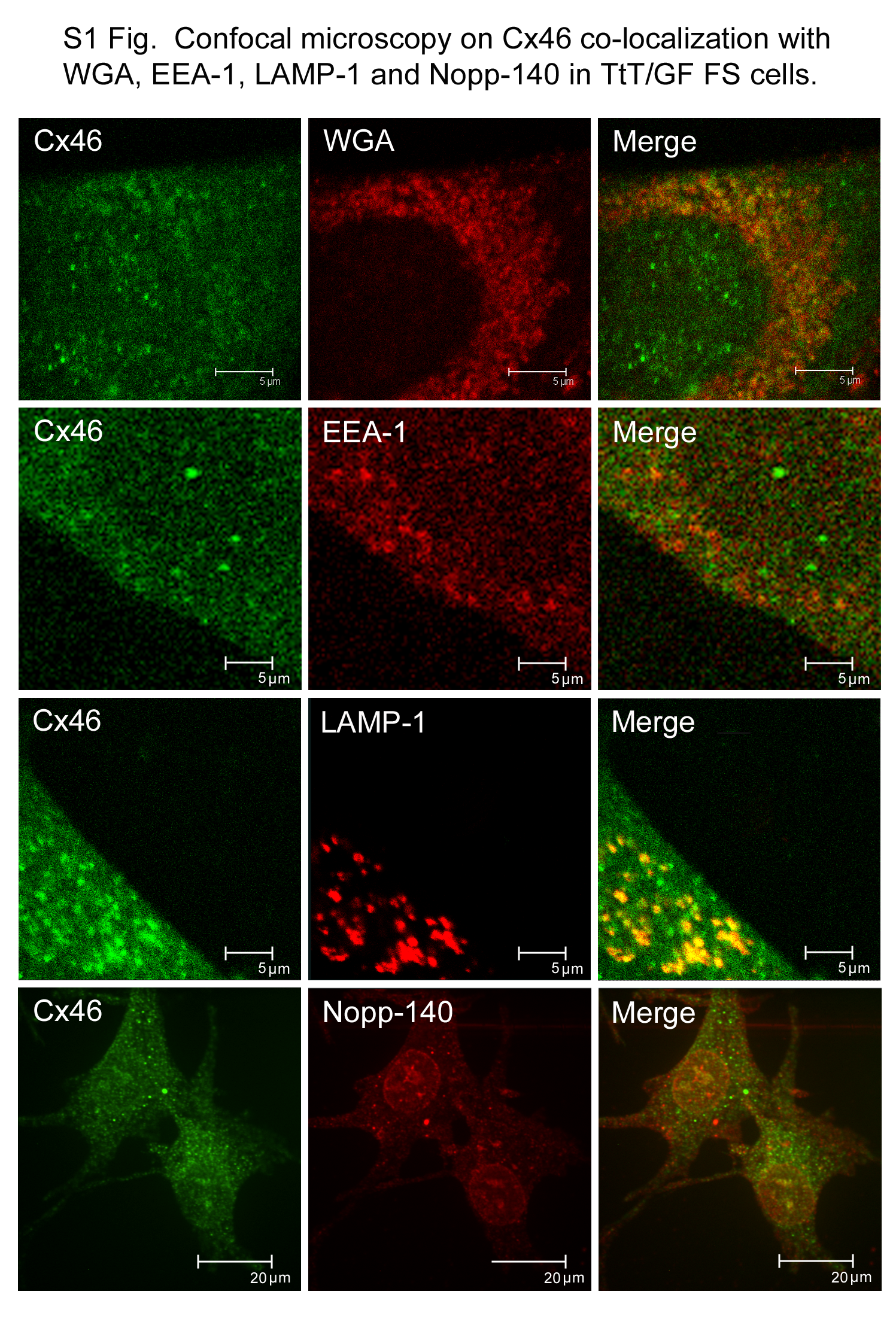

Supplement: S1 Fig — (TIF) [file pone.0182495.s001.tif]

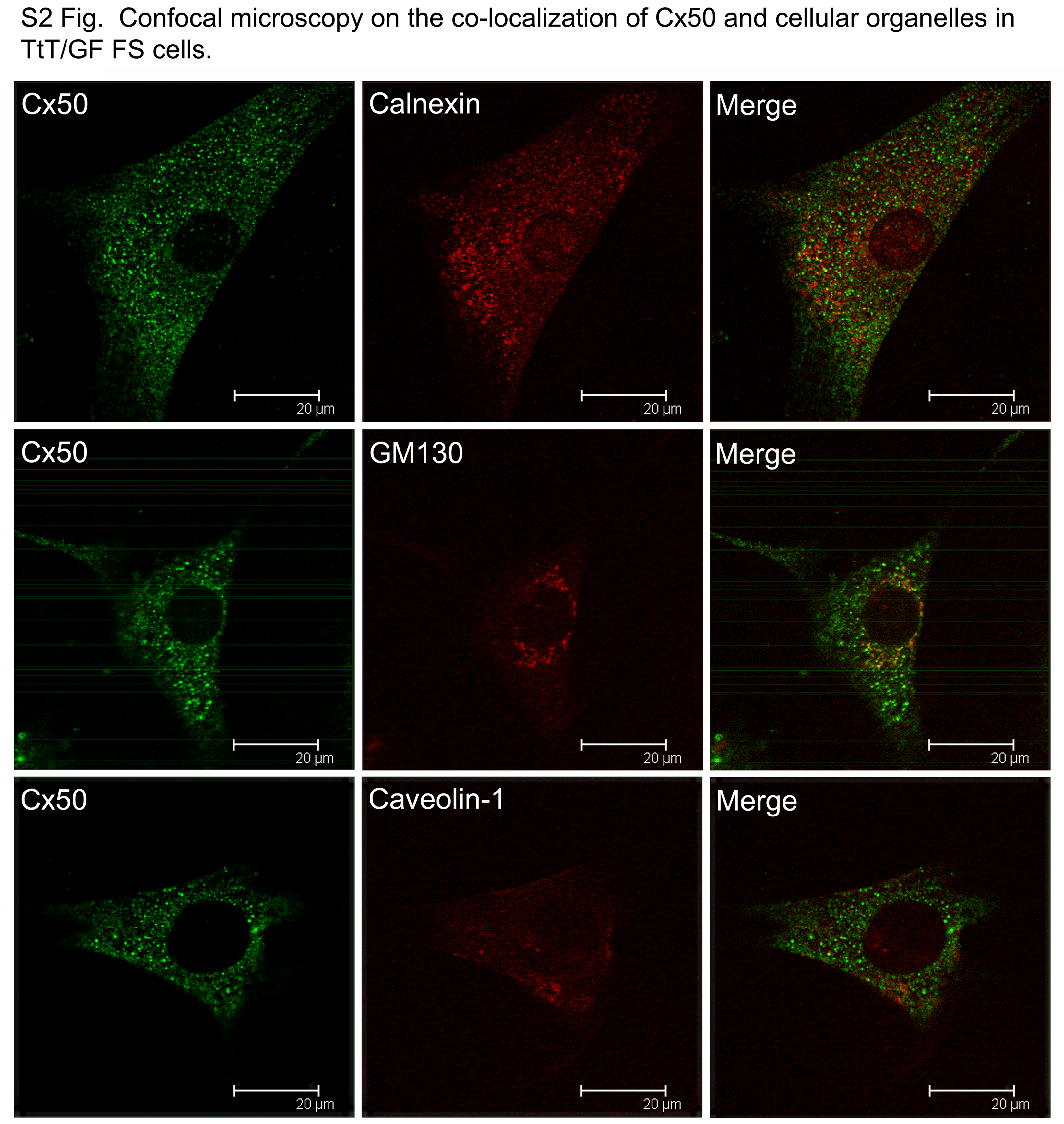

Supplement: S2 Fig — (TIF) [file pone.0182495.s002.tif]

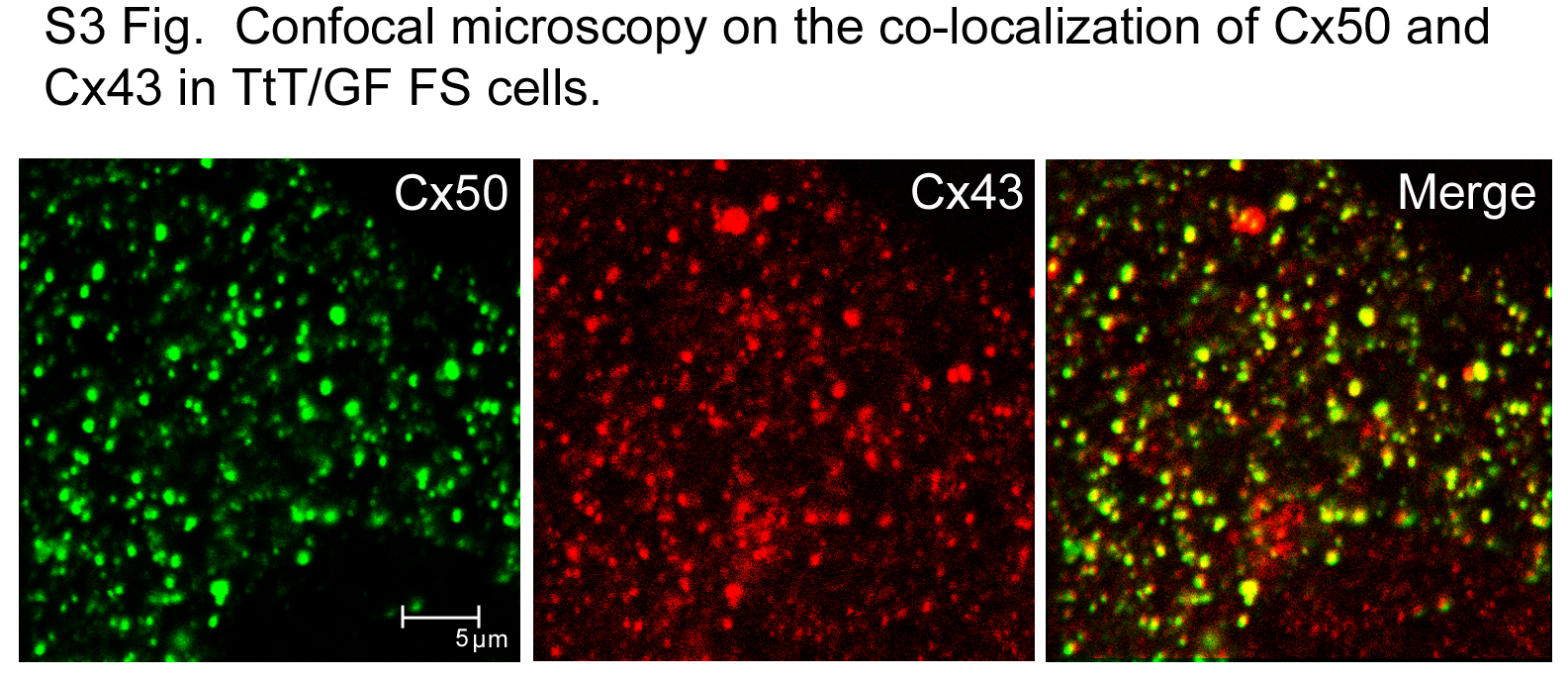

Supplement: S3 Fig — (TIF) [file pone.0182495.s003.tif]
